# ExonSurfer: a web-tool to design primers at exon–exon junctions

**DOI:** 10.1186/s12864-024-10456-2

**Published:** 2024-06-12

**Authors:** Pablo Monfort-Lanzas, Elena Cristina Rusu, Lucia Parrakova, Cornelia A. Karg, Dorina-Elina Kernbichler, Dietmar Rieder, Peter Lackner, Hubert Hackl, Johanna M. Gostner

**Affiliations:** 1 Institute of Medical Biochemistry, Biochemical Immunotoxicology Group, Biocenter, Medical University of Innsbruck, Innrain 80, 6020 Innsbruck, Austria; 2 Institute of Medical Biochemistry, Core Facility Metabolomics II, Biocenter, Medical University of Innsbruck, Innrain 80, 6020 Innsbruck, Austria; 3 Institute of Bioinformatics, Biocenter, Medical University of Innsbruck, Innrain 80, 6020 Innsbruck, Austria; 4 SeqPlexing SL, Valencia, Spain; 5 Institute of Integrative Systems Biology (I2Sysbio), University of Valencia and Consejo Superior de Investigaciones Científicas (CSIC), Valencia, Spain; 6 Department of Biosciences and Medical Biology, University of Salzburg, 5020 Salzburg, Austria

**Keywords:** Exon-exon junction, Primer design, RT-qPCR, Alternative splicing, Gene expression, Web tool

## Abstract

**Background:**

Reverse transcription quantitative PCR (RT-qPCR) with intercalating dyes is one of the main techniques to assess gene expression levels used in basic and applied research as well as in diagnostics. However, primer design for RT-qPCR can be complex due to the high demands on primer quality. Primers are best placed on exon junctions, should avoid polymorphic regions, be specific to the target transcripts and also prevent genomic amplification accurately, among others. Current software tools manage to meet all the necessary criteria only insufficiently. Here, we present ExonSurfer, a novel, user-friendly web-tool for qPCR primer design.

**Results:**

ExonSurfer combines the different steps of the primer design process, encompassing target selection, specificity and self-complementarity assessment, and the avoidance of issues arising from polymorphisms. Amplification of potentially contaminating genomic DNA is avoided by designing primers on exon-exon junctions, moreover, a genomic alignment is performed to filter the primers accordingly and inform the user of any predicted interaction. In order to test the whole performance of the application, we designed primer pairs for 26 targets and checked both primer efficiency, amplicon melting temperature and length and confirmed the targeted amplicon by Sanger sequencing. Most of the tested primers accurately and selectively amplified the corresponding targets.

**Conclusion:**

ExonSurfer offers a comprehensive end-to-end primer design, guaranteeing transcript-specific amplification. The user interface is intuitive, providing essential specificity and amplicon details. The tool can also be used by command line and the source code is available. Overall, we expect ExonSurfer to facilitate RT-qPCR set-up for researchers in many fields.

**Supplementary Information:**

The online version contains supplementary material available at 10.1186/s12864-024-10456-2.

## Background

Accurate detection and quantification of gene expression levels is crucial for understanding biological processes and disease mechanisms. Intercalating dye-based reverse transcription qPCR (RT-qPCR) is a widely used method for gene expression analysis, and designing primers that span exon-exon junctions is critical for specific and accurate transcript detection [1]. However, designing such primers is a challenging task, as it requires careful consideration of the transcript specificity, the exon junction, primer length and melting temperature, or the avoidance of common single nucleotide polymorphism (SNPs) in the primer sequence [2]. With so many factors to take into account, primer design is a commonly automated task for which multiple tools are available [3]. Primer3 is the most widely used primer design tool, both directly (using the command line, Python package or web tools) and indirectly, by accessing one of the multiple applications that are based on it [4–6]. Although Primer3 is capable of handling most primer design challenges in the hands of an expert, it requires extensive experience and the use of additional tools to extract the target sequence or check for specificity. Primer-BLAST’s main focus is specificity to the target [7]. Furthermore, there is no option to perform a genomic alignment. Even though the user can choose to only design primers on exon junctions, this is often not enough to ensure the absence of genomic interference during qPCR. In addition, it presumably leverages Primer3’s built-in capability to detect dimers or hairpins, and there is extensive room for improvement in this area [8]. Other more RT-qPCR-focused tools include Ex-Ex-Primer [9] QuantPrimer [10], IDT’s private software (https://eu.idtdna.com/scitools/Applications/RealTimePCR) and the highly versatile FastPCR [11]. While Ex-Ex-Primer assists a wide variety of qPCR setups, it requires the user to input the target junction, thus entailing the need for a prior multiple sequence alignment in the case of genes with several transcripts. In addition, it does not avoid SNPs or other variants and doesn’t allow the user to detect multiple transcripts from the same gene with one primer pair. IDT’s software is straightforward and easy to use, but lacks documentation and specificity results, among others. FastPCR supports a wide range of primer design applications, but it requires the user to insert the sequence, it works exclusively on Microsoft Windows machines and needs to be installed.

**Fig. 1:**
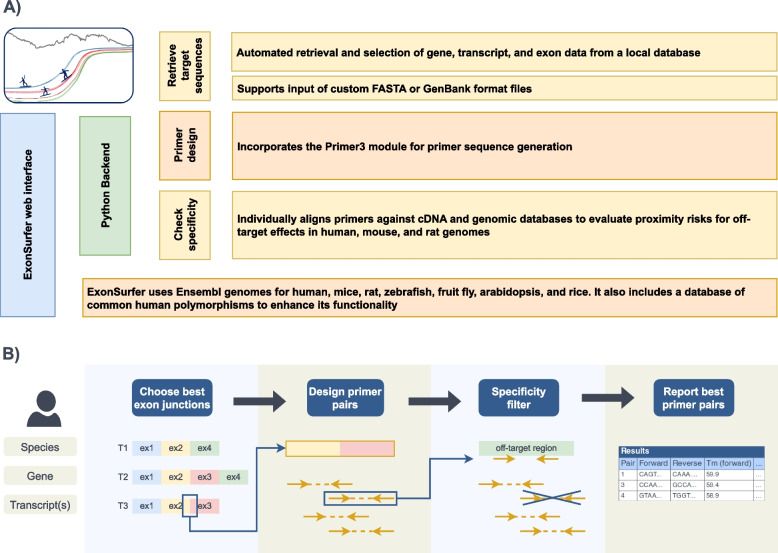
**A** Schematic representation of ExonSurfer’s interface and backend, detailing the automated retrieval of genetic information, integration of custom sequence files, and application of the Primer3 module for primer design, along with specificity checks against genomic databases to preclude off-target effects in model organisms. **B** Diagram illustrating the user-directed primer design workflow, from exon junction selection to the final reporting of optimal primer pairs, all while employing specificity filters to ensure high fidelity in downstream qPCR applications

In order to have a single resource, which overcomes these limitations, we developed ExonSurfer. Multiple applications and databases for robust primer design support are combined in ExonSurfer (Fig. 1A). This user-friendly web-based tool streamlines the different steps of primer design for RT-qPCR with intercalating dyes (Fig. 1B), targeting genes of different organisms (*Homo sapiens*, *Mus musculus, Rattus norvegicus, Danio rerio, Arabidopsis thaliana, Oryza sativa and Drosophila melanogaster*. ExonSurfer enables users to design primers that avoid common polymorphisms, checks for specificity and self-complementarity, and can guarantee the span of exon-exon junctions. Furthermore, users can select specific splice variants and customize primer length, thermal parameters, and maximum amplicon length to best suit their amplification procedure. ExonSurfer efficiently designs primers that meet these requirements, reducing the need for extensive in silico and in vitro optimization or the need to use several tools. For the assessment of ExonSurfer, we chose 26 targets needed in ongoing projects, ensuring a broad representation across various gene sizes, expression levels, and transcript variants. This primer selection was designed and verified by qPCR to provide an objective evaluation of ExonSurfer’s capabilities. Then we checked the predicted amplicon size of each primer pair against the actual PCR product size. In addition, the PCR products were subjected to Sanger sequencing to confirm their accuracy. Most of the amplified amplicons were confirmed to be accurate through this thorough validation process, without the need for further qPCR optimization steps.

## Implementation

### Programming language and software packages

ExonSurfer is an open-source web-tool developed using Python and the Django framework. The tool uses PyEnsembl (github.com/openvax/pyensembl) to access the Ensembl database (release 108 for animals and release 57 for plants) [12], primer3-py (libnano.github.io/primer3-py) and BLAST + software packages [13] to design and filter primer pairs based on their specificity and self-complementarity, among other parameters.

### Data sources

ExonSurfer is currently available for these species: *Homo sapiens*, *Mus musculus* *,* *Rattus norvegicus, Danio rerio* *,* *Oryza sativa, Arabidopsis thaliana and Drosophila melanogaster*. The gene and transcript information used by ExonSurfer is retrieved from the Ensembl database (release 108 for the animal species, release 57 for the plant species). In the case of *Homo sapiens*, the dbSNP database [14] was previously downloaded and used to mask all common SNPs (minor allele frequency % in any population and study), so, by default, primers are not placed on SNPs or other short variants, though this option can be disabled. To assess primer specificity, ExonSurfer performs two sequential BLAST alignments against the cDNA and genomic databases from Ensembl. For *Homo sapiens*, the database is enriched with the protein coding mRNA from RefSeq [15], which increases the specificity and sensitivity of the primer design process. By performing these alignments, ExonSurfer is able to identify and filter out primer pairs with potential off-target hits, including those against other genes and non-targeted transcript isoforms from the target gene itself.

### Workflow

ExonSurfer’s workflow (Fig. 1B) consists of the following steps: (i) it selects the best exon junctions based on the transcripts selected by the user, (ii) it designs multiple primer pairs for each junction, and (iii) it filters these primer pairs based on their specificity and self-complementarity. Finally, it ranks and reports the remaining primer pairs.

### Junction and sequence selection

A key feature of ExonSurfer is the ability to select the best exon junctions for the user’s selected targets automatically. The user can choose one or more transcript isoforms, and ExonSurfer will select the junctions that are present in all the chosen targets and absent in all the unselected transcripts. In cases for which the user’s specified criteria cannot be met, ExonSurfer’s algorithm selects the junctions that mostly meet the user’s criteria and informs the user of any possible non-targeted or off-target transcript isoforms. In these suboptimal cases, the canonical transcript is targeted preferentially. Additionally, for the human genome, the user can choose to design the primers on a pre-masked genome, thereby avoiding areas with high genetic variation during the primer design process.

Transcript sequences in GenBank or FASTA format can also be used as input for novel transcript discovery. If the origin is one of the available species, ExonSurfer will also perform specificity testing on them. For the FASTA file, the exon junctions placement in the sequence should be marked in the header.

### Primer design

ExonSurfer uses primer3-py in order to design primer pairs, placing one primer’s 3’end in the exon junction. Moreover, ExonSurfer also allows the user to design primers flanking exon junctions rather than spanning them.

### Specificity filter

ExonSurfer checks specificity in two distinct steps: for the first round it assesses alignments against the mRNA, since this will be the main form of nucleic acids present in the reaction. After performing the BLAST alignments, primer pairs are annotated for possible off-target products considering both other genes and non-targeted transcript isoforms from the target gene. ExonSurfer performs the second BLAST round against the whole genomic DNA with the best primers from the previous step. In addition, unproductive off-target alignments (that is, single primer alignments) are stored for further filtering.

### Web interface

**Fig. 2:**
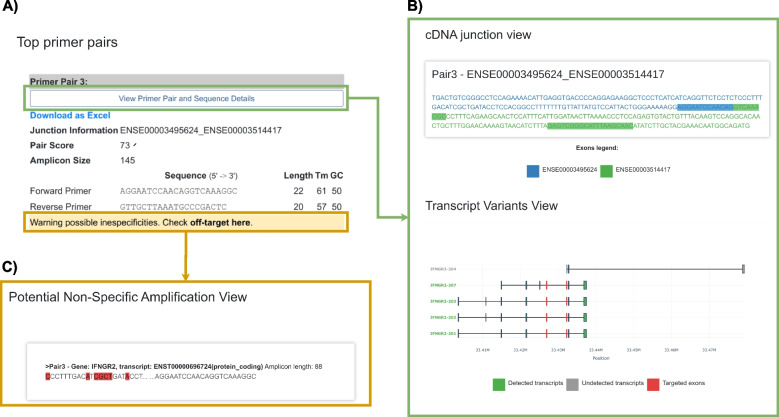
**A** Results view from ExonSurfer, detailing top primer pairs with scores and sequences, and highlighting specificity warnings. **B** cDNA view showing primer binding on differentially colored exons, with primer efficacy on various transcripts indicated in green for positive and gray for negative detections, and red for the targeted exon. **C** Potential off-target amplification regions are shown, with primer mismatches highlighted in red, facilitating off-target assessment

ExonSurfer is provided as a web service accessible through a web browser without the need for additional software or registration. The web interface (Fig. 2) is designed to be user-friendly and intuitive. On the main page, users are presented with a form to input search parameters such as species and gene symbol. The interface also allows for the uploading of custom sequence data in GenBank or FASTA file formats, facilitating the detection of novel transcripts. Users have the option to design primers that span or flank exon-exon junctions, tailored to their experimental requirements. Primer parameters like length, GC content, melting temperature, and maximum amplicon length can also be specified.

Upon submitting the search parameters, ExonSurfer initiates the primer design process. The output page (Fig. 2A) provides a comprehensive summary, displaying details such as the number of primer pairs generated for each selected junction and their specificity information. Each primer pair’s details, including the amplicon sequence (Fig. 2B) and BLAST alignment information (Fig. 2C), are available for review. Additionally, the interface enables the download of results in various table formats like CSV or Excel.

The results are stored for a predetermined duration and can be accessed using the link provided upon submission.

### Primer validation

In brief, total RNA was isolated using the RNeasy Mini Kit (Qiagen, Germany) from four cell lines: monocytic leukemia THP-1 cells (CLS, Eppelheim, Germany), transformed bronchial epithelium Beas-2B cells (ATCC-CRL-9609) (LGC Standards GmbH, Wesel, Germany), the telomerase-immortalized human corneal epithelial cell line hTCEpi (Evercyte, Vienna, Austria), and the telomerase-immortalized human corneal endothelial cell line HCEnC-21 T (kindly provided by Prof. U. Jurkunas, Boston, MA, US) [16]. THP-1 cells and Beas-2B cells were cultured in RPMI 1640 medium (Sigma-Aldrich, Vienna, Austria) supplemented with 10% fetal calf serum (FCS, Gibco™, Life Technologies, Carlsbad, California, U.S.). hTCEpi cells were cultured in growth factor supplemented keratinocyte growth medium 2 medium (C-20011, Promocell, Heidelberg, Germany) and HCEn-21 T cells were maintained in complete medium (OptiMEM-I®, ThermoFisher Scientific, Vienna, Austria) supplemented with 8% FCS, 5 ng/mL epidermal growth factor (Peprotech, London, UK), 52 ng/mL pituitary extract (Promocell, Heidelberg, Germany), 200 µg/mL calcium chloride (Sigma-Aldrich, Vienna, Austria), and 0.8 mg/mL chondroitin sulfate (Sigma-Aldrich, Vienna, Austria). Prior to RNA isolation, cell lysates were homogenized using the QIAshredder system (Qiagen, Germany). RNA isolation was performed with the RNeasy Mini kit (Qiagen, Germany). RNA quantification and assessment of purity (260/280 nm) was performed using a Nanodrop spectrophotometer (ThermoFisher, Whaltham, MA, US). No further DNase treatment was performed.

Single-stranded cDNA was synthesized from 1 μg of total RNA using the LunaScript RT SuperMix (New England BioLabs, Frankfurt am Main, Germany) according to the manufacturer’s instructions. For each primer pair, the efficiency was determined. To evaluate the performance of a primer set, a serial dilution of cDNA (50 ng, 25 ng, 12.5 ng, 6.25 ng, 3.125 ng and 1.6 ng of cDNA) was performed and added to 50 nM of specific primers and the Luna Universal Probe qPCR Master Mix (New England BioLabs, Germany). Total reaction volume was 10 μl. Reactions were performed in duplicates. qPCR was performed using the Rotor-Gene-Q 5plex HRM (Qiagen, Hilden, Germany) under the following conditions: initial denaturation at 95 °C for 60 s; 40 cycles: 95 °C for 15 s, 60 °C for 30 s (fluorescence acquisition); melt curve analysis: ramp from 60 to 95 °C (rising by one degree each step); 25 °C for 60 s and hold on room temperature. For each primer pair, the amplicon length was verified once by gel electrophoresis and the PCR product was sent to sequencing (Microsynth, Balgach, Switzerland). Ct values were plotted against the natural logarithm of the template concentration and PCR efficiencies were calculated from the slope of the regression line according to the equation E = 10^[−1/slope]^ [17–19].

## Results and discussion

In the past year, the primary focus of the scientific community has been on enhancing the methodology for analyzing qPCR data [20, 21] and numerous methods have been proposed to increase the statistical robustness of the analysis [22–24]. However, there is still a need to refine the primer design process to improve the overall analysis.

ExonSurfer represents a novel, integrated approach in the realm of RT-qPCR primer design, particularly regarding primers spanning exon-exon junctions. This tool, developed for intercalating dye-based qPCR, offers accuracy and specificity in its function. By adjusting the user-parameters, ExonSurfer can easily adapt to several types of primer design focused on exon junctions, including RT digital PCR (dPCR) based on EvaGreen dye. It operates independently of external software resources, as depicted in Fig. 1, highlighting its unique capacity for autonomous, end-to-end primer design. ExonSurfer follows the FAIR principles [25].

First of all, ExonSurfer identifies the optimal exon junctions for the selected targets. In cases where no perfect junctions are available (i.e. there are no junctions exclusively present in the selected transcripts), our approach shifts to selecting junctions that are present across all targets, preferentially covering Ensembl’s canonical isoform. Any untargeted transcripts are reported as off-target products. For the human genome, primers are configured to avoid SNPs with a minor allele frequency (MAF) larger than 1%, thereby mitigating the potential of uneven amplification efficiency in different samples. Afterwards, we design primers that span exon junctions by a minimal number of bases in the 3’ end and the 5’ end. ExonSurfer also allows the user to design primers flanking exon junctions rather than spanning them. However, this option is only recommended for experiments where genomic contamination is not a concern.

Once multiple primer pairs are designed for a set of targets, we align them against the corresponding mRNA and assess possible off-target formation. This is especially concerning because off-target presence leads to false positives in RT-qPCR experiments. We keep the most specific primer pairs and align them against the genomic DNA; even though primers are designed on exon junctions, that sequence might still be present in the genomic DNA in any other location, so an additional alignment step is necessary. If all the primer pairs have off-targets, we report them so the user can make an informed decision (Fig. 2C). Overall, this comprehensive primer information makes it easy for researchers to select the most appropriate primer pair for their specific experimental conditions.

In this study, we validated the functionality, performance, and efficiency of ExonSurfer by designing primer pairs for 26 different genes. These genes were selected as a real world case, due to their implication in several ongoing projects. A summary of the most representative results is listed in Table 1, highlighting key findings such as primer efficiency and specificity. Complete information on the primer sequences, design parameters, and comprehensive results across all tested cell lines can be found in Additional file 1.

**Table 1:** Summary of primer efficacy for selected genes across various cell lines. Primer pairs that specifically and correctly amplified the target are indicated in **bold**

Gene	Forward Primer	Reverse Primer	Predicted Amplicon Tm (°C)	Primer Efficency	R^2^	Cell Line	ExonSurfer Predicted Off-target	Successful Sequencing
**ACTB**	AGTCATAGTCCGCCTAGAAGC	GATCAAGATCATTGCTCCTCCTG	88	2.07	0.997	THP-1	Yes	Yes
**ADH5**	ATCAGCTCCACTCAGGGTATAG	GAACATGGCGAACGAGGTTATC	87	2.18	0.999	THP-1	No	Yes
**ALDH2**	CATCATGTCAGATGCCGATATGGATTG	CTCGGTCTTGCTATCAAAGGGG	90	2.03	0.999	THP-1	Yes	Yes
**ATP1A1**	ACTTGAGCCGGGGATTAACATC	CACACCCAGGTACAGATTATCG	87	2.15	0.996	HCEN-21 T	No	Yes
**ATP1A3**	AAACAGCCCGAAGATCAGG	AGCAGTGGACATACGAGCAG	87	2.06	0.984	HCEN-21 T	Yes	Yes
**B2M**	AGTTAAGTGGGATCGAGACATG	CCTGCTCAGATACATCAAACATGG	81	2.36	0.987	THP-1	No	Yes
**CA2**	GCAAACACAACGGACCTGAG	AATCTGTAAGTGCCATCCAGGG	87	2.10	0.996	HCEN-21 T	Yes	Yes
**DHFR**	AACTGCCACCAACTATCCAGAC	TCAGCAGAGAACTCAAGGAACC	81	2.50	0.994	THP-1	Yes	Yes
**ESD**	GTGCAAGTTAAACCTGAGAGCC	CACCGTAGAATCGCCTACCATTTG	83	2.18	0.993	THP-1	No	Yes
GAPDH	ATCTTCTTTTGCGTCGCCAG	TGTAAACCATGTAGTTGAGGTCAATG	86	2.09	0.990	THP-1	Yes	Yes
**HPRT1**	CCTGGCGTCGTGATTAGTGATG	ACAGAGGGCTACAATGTGATGG	83	2.18	0.999	THP-1	No	Yes
IFNGR1	TAACACCATAGTTCTTTACCTCTACGG	TCTCCTACCCCTTGTCATGCAG	86	2.12	0.998	THP-1	No	Yes
**IFNGR2**	CCGACAGTAAATGGTTCACGGC	CCGACAGTCACATTCCGATAGTG	87	2.17	0.998	THP-1	No	Yes
KRT12	CTCCCCGCTCTTTTCAATGAAC	AAGAAGAACCACGAGGATGAGC	89				No	no PCR amplicon
**KRT14**	TCTCCACATTGACATCTCCACC	TTCCGCACCAAGTATGAGACAG	89	2.02	0.999	hTCEpi	No	Yes
**KRT16**	CTCCTCGGTCTTGCTCAGGAAC	GATGTGAACGTGGAGATGGATG	88	2.02	0.996	hTCEpi	Yes	Yes
KRT3	ATGATCTCGCTCTTGGTATTTC	CTCTACGACGCTGAGCTATC	88				No	no PCR amplicon
**KRT5**	CAGCTCCGCATCAAAGAACATC	ATCAACAAGCGTACCACTGC	83	2.04	0.992	hTCEpi	Yes	Yes
**MTHFR**	CATCCGGTCAAACCTTGAGATG	GATAGTTCGAGATGTTCCACCC	88	2.42	0.995	THP-1	Yes	Yes
RPL37A	CCTGGACGTACAATACCACTTC	ACTGAATTACAATAATCCATCTTGGC	82	2.37	0.981	THP-1	Yes	Yes
RPLP0	GCTCCCACAATGAAACATTTCG	TCCTCGTGGAAGTGACATCGTC	85	2.15	0.992	THP-1	Yes	Yes
**SHMT1**	TATTCCAGGTTTCGGGAGTAGC	ATCTATGCCCTACAAGGTGAACCC	85	2.24	0.990	Beas2B	No	Yes
**SHMT2**	TCCTGGCAAGAGATACTATGGG	ATGATCCGGTCGTGAGGTTG	90	2.19	0.992	THP-1	No	Yes
**SLC9A1**	TGACAAGTAGGCCATGTAGCTG	CGTCACTGTGGTCCTGTATCAC	90	2.20	0.994	HCEN-21 T	No	Yes
**TBP**	ACTCACAGACTCTCACAACTGC	CAAACCGCTTGGGATTATATTCGG	87	2.22	0.983	THP-1	Yes	Yes
**TJP1**	GCTGAAGGACTCACAGGAATAG	GTGAAATCGCACAGTTTGGCAC	81	2.21	0.997	HCEN-21 T	Yes	Yes

**Fig. 3:**
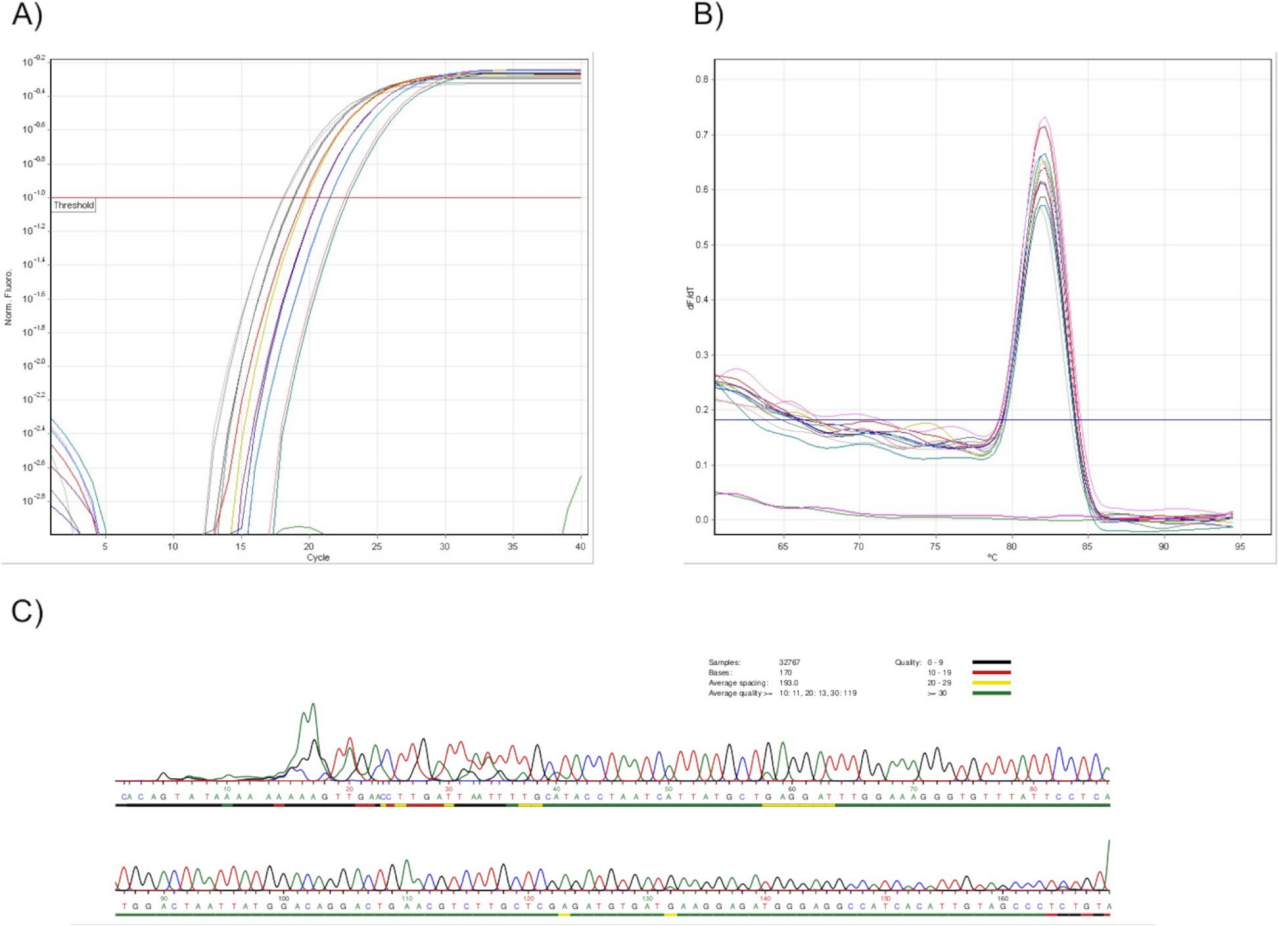
Results from qPCR amplification using ExonSurfer-designed primers for the HPRT1 gene, tested in Beas-2B cells. **A** qPCR SYBR Green I fluorescence history versus cycle number of target gene in serial dilutions of cDNA. The crossing points of threshold fluorescence (Ct values) are determined. Threshold fluorescence is defined as the point at which the fluorescence rises appreciably above the background fluorescence. **B** Melting curves illustrate the dissociation behavior of the amplified products, with a peak melting temperature (Tm) at 82.0 °C, indicative of primer specificity. **C** Sequencing quality of the HPRT1 amplicon as determined by Sanger sequencing, demonstrating the fidelity and accuracy of the amplified product

We tested several primer pairs in the wet lab, using RNA extracted from a diverse set of cell lines: THP-1, Beas-2B, hTCEpi, and HCEnC-21 T. We assessed both the specificity and efficiency of these primers. From the 26 primer pairs tested, 24 were able to efficiently amplify a target (E ≥ 2) in at least one cell line. However, two of them, targeting KRT3 and KRT12 genes, were unable to amplify the sequence, presumably due to the low levels of transcript expression in the tested cell lines. Additionally, 20 primer pairs obtained a coefficient of determination (R^2^) > 0.99 with at least one RNA extract, indicating optimal performance. Furthermore, 20 primer pairs were confirmed to amplify the target specifically and correctly, as verified by sequencing. The relevant primer and experimental setup information is detailed in the method description as well as in the supplementary files provided with this manuscript, addressing transparency and reproducibility concerns. As an example of these findings, the results from the HPRT1 primer pair can be checked in Fig. 3.

Notably, as initially suggested by ExonSurfer predictions, we encountered several challenges with primer specificity and off-target amplification. Specifically, GAPDH primers showed suboptimal performance in the wet lab experiments, demonstrating off-target amplification. ExonSurfer had identified potential off-targets for GAPDH, involving transcripts ENST00000396861 and ENST00000492719. Additionally, ExonSurfer predicted potential nonspecific off-target amplification for the RPL37A primers due to genomic DNA nonspecificity, which was in line with the wet lab qPCR results. RPLP0's primers, as indicated by ExonSurfer, showed potential nonspecific amplification and off-target effects related to the RPLP0P2 gene. Furthermore, sequencing of IFNGR1 encountered issues, indicative of primer inefficiency or nonspecific binding, which could be attributed to the potential amplification of other transcripts from the same gene. The preliminary validation of our web-based tool, reveals variable success rates, reflecting the unpredictable nature of primer design. These findings align with the results reported by Andreson et al. [26] and Cordaro and colleagues [27], accentuating the complexity inherent in primer specificity analyses.

To summarize, validation experiments demonstrated that most of the designed primers had high accuracy and specificity, producing PCR products with high efficiency, and sequencing data confirmed the identity of the targeted transcripts. The amplification plots, melting curves and gel electrophoresis results for all the targets tested in the four cell lines can be found in the Additional file 2, and Additional file 3.

One of the main limitations of ExonSurfer is the range of species that the tool currently supports. Although the tool has been validated for *Homo sapiens*, further development and experiments are needed for other species. Additionally, while ExonSurfer uses the Ensembl database for gene and transcript information, databases such as RefSeq are not included in the main pipeline. This issue is at least partially resolved by the possibility to upload custom GenBank or FASTA files for primer design expands the tool’s usability, providing researchers with greater flexibility in designing primers for their specific targets. In the context of ExonSurfer’s application domain, it is pertinent to note that its algorithmic framework is precisely tailored for the design of primers spanning exon-exon junctions. This fact, while beneficial for its target specificity and efficiency, inherently does not extend to methodologies such as those required for amplifying low abundance transcripts or analyzing degraded RNA samples, which may necessitate alternative primer design strategies.

In future versions, we plan to expand the tool’s capabilities by adding support for additional species and databases, as well as including other primer design methods to improve its versatility.

## Conclusion

ExonSurfer is an end-to-end application for RT-qPCR primer design based on intercalating dies. The tool’s high accuracy and specificity make it a valuable asset in gene expression analysis, particularly in studies involving complex transcript variants. ExonSurfer provides researchers a powerful and efficient solution for designing primers that meet all the necessary requirements for RT-qPCR-based gene expression analysis.

## Availability and Requirements

Project name: ExonSurfer.

Project home page: https://exonsurfer.i-med.ac.at/

Operating system(s): User interface: Platform independent; Server side: Linux.

Programming language: Django, SQLite, HTML, JavaScript, and Python.

Other requirements: Web browser (supporting JavaScript).

License: Specified on the GitHub repository.

Any restrictions to use by non-academics: No restrictions for non-commercial use.

### Supplementary Information

Additional file 1: Supplementary Table 1 displays primer designs generated using ExonSurfer, including gene details, efficiency percentages, tested cell lines, primer sequences, amplicon characteristics, melting temperatures, alignment scores, and transcript detection outcomes. Primer efficiency is calculated by the equation E = 10-1/slope, with an efficiency of 2 indicating that for each cycle the amount of product doubles.

Additional file 2: Supplementary File 2 presents qPCR data for 24 targets in Beas-2B and THP-1 cells, detailing fluorescence intensities, melting curves, Ct versus cDNA concentration graphs, and summarizes efficiency metrics for each primer pair.

Additional file 3: Supplementary File 3 displays electrophoresis gels containing the agarose gel-separated amplicons from the PCR products of the validation genes.

## Data Availability

The ExonSurfer tool and all data generated or analyzed during its ongoing development are accessible via the provided web tool link. ExonSurfer is accessible through a web browser with no login requirement. The tool is freely available and can be accessed at https://exonsurfer.i-med.ac.at/ (source code available at github.com/pamonlan/ExonSurferWeb). In addition, a Python package (github.com/CrisRu95/ExonSurfer) can be run from the command line, as well as integrated into existing bioinformatics pipelines. This makes it highly flexible and enables bioinformatic experts to customize the primer design process according to their needs. Moreover, the tool is available as a Docker image, which makes it easy to run on any system without the need for complex installation procedures. The databases used for sequence extraction and BLAST alignment are available in zenodo, 10.5281/zenodo.7638572.

## Abbreviations

BLAST: Basic Local Alignment Search Tool
Ct: Cycle Threshold
FCS: Fetal Calf Serum
MAF: Minor Allele Frequency
PCR: Polymerase Chain Reaction
qPCR: Quantitative PCR
RT-qPCR: Reverse Transcription Quantitative PCR
SNP: Single Nucleotide Polymorphism
Tm: Melting Temperature
