# Supplementary material for: ExonSurfer: a web-tool to design primers at exon–exon junctions
Source: BMC Genomics. 2024 Jun 12;25:594. doi: 10.1186/s12864-024-10456-2 (PMC11170769; doi:10.1186/s12864-024-10456-2)
Supplement: Supplementary file 2 — Additional file 2: Supplementary File 2 presents qPCR data for 24 targets in Beas-2B and THP-1 cells, detailing fluorescence intensities, melting curves, Ct versus cDNA concentration graphs, and summarizes efficiency metrics for each primer pair. [file 12864_2024_10456_MOESM2_ESM.pptx]

## Slide 1
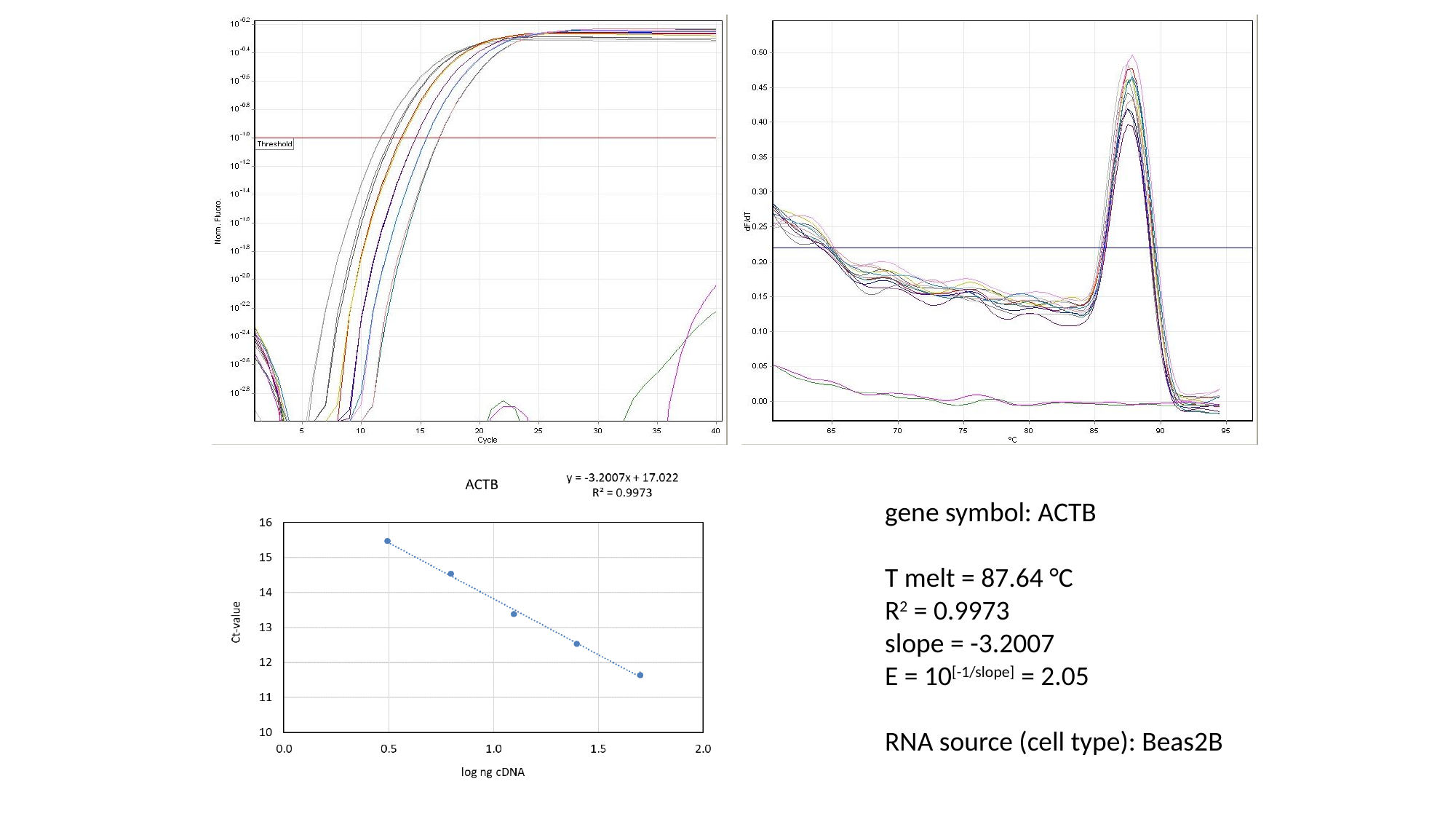

gene symbol: ACTB
T melt = 87.64 °C
R2 = 0.9973
slope = -3.2007
E = 10[-1/slope] = 2.05
RNA source (cell type): Beas2B

## Slide 2
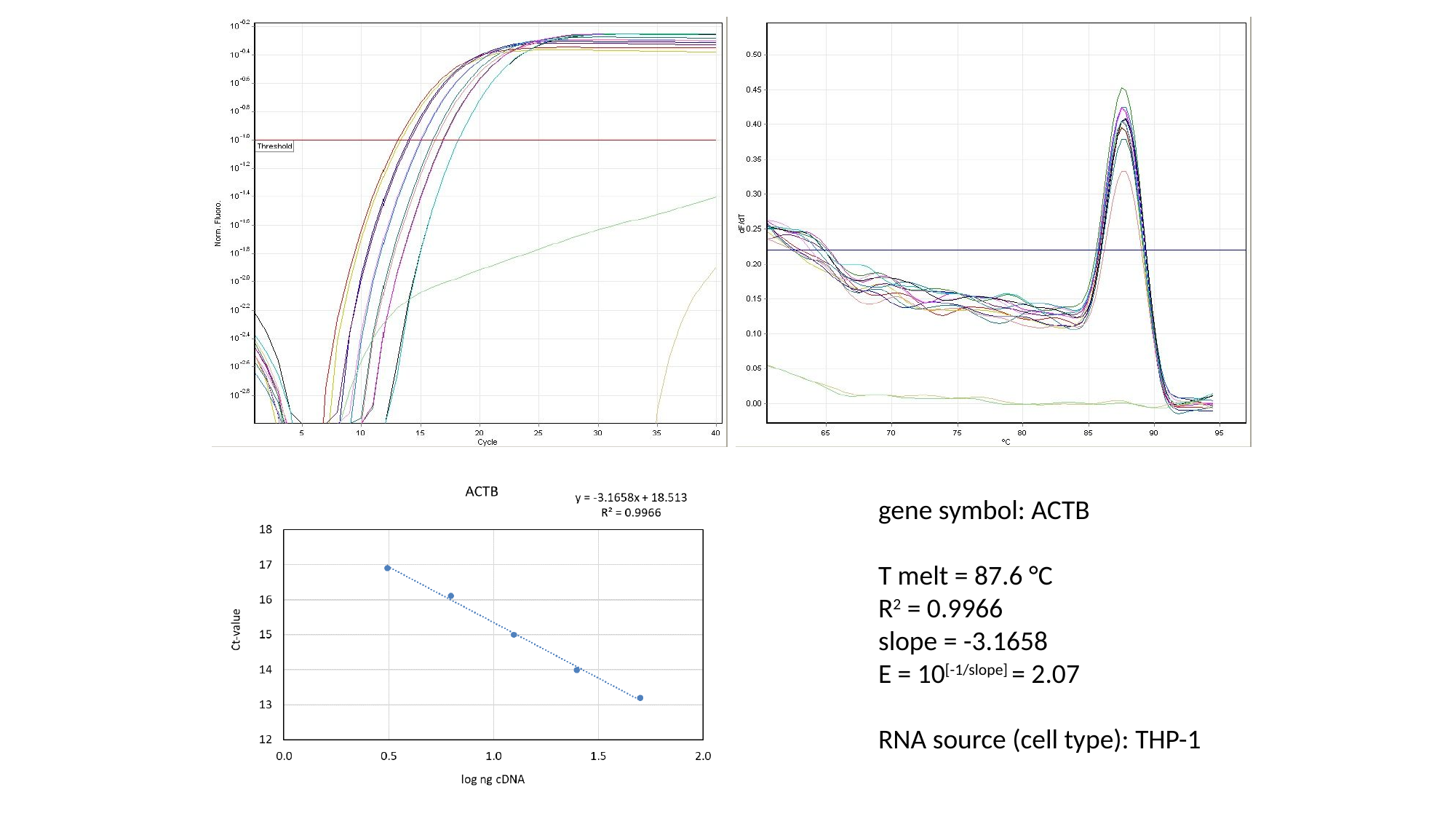

gene symbol: ACTB
T melt = 87.6 °C
R2 = 0.9966
slope = -3.1658
E = 10[-1/slope] = 2.07
RNA source (cell type): THP-1

## Slide 3
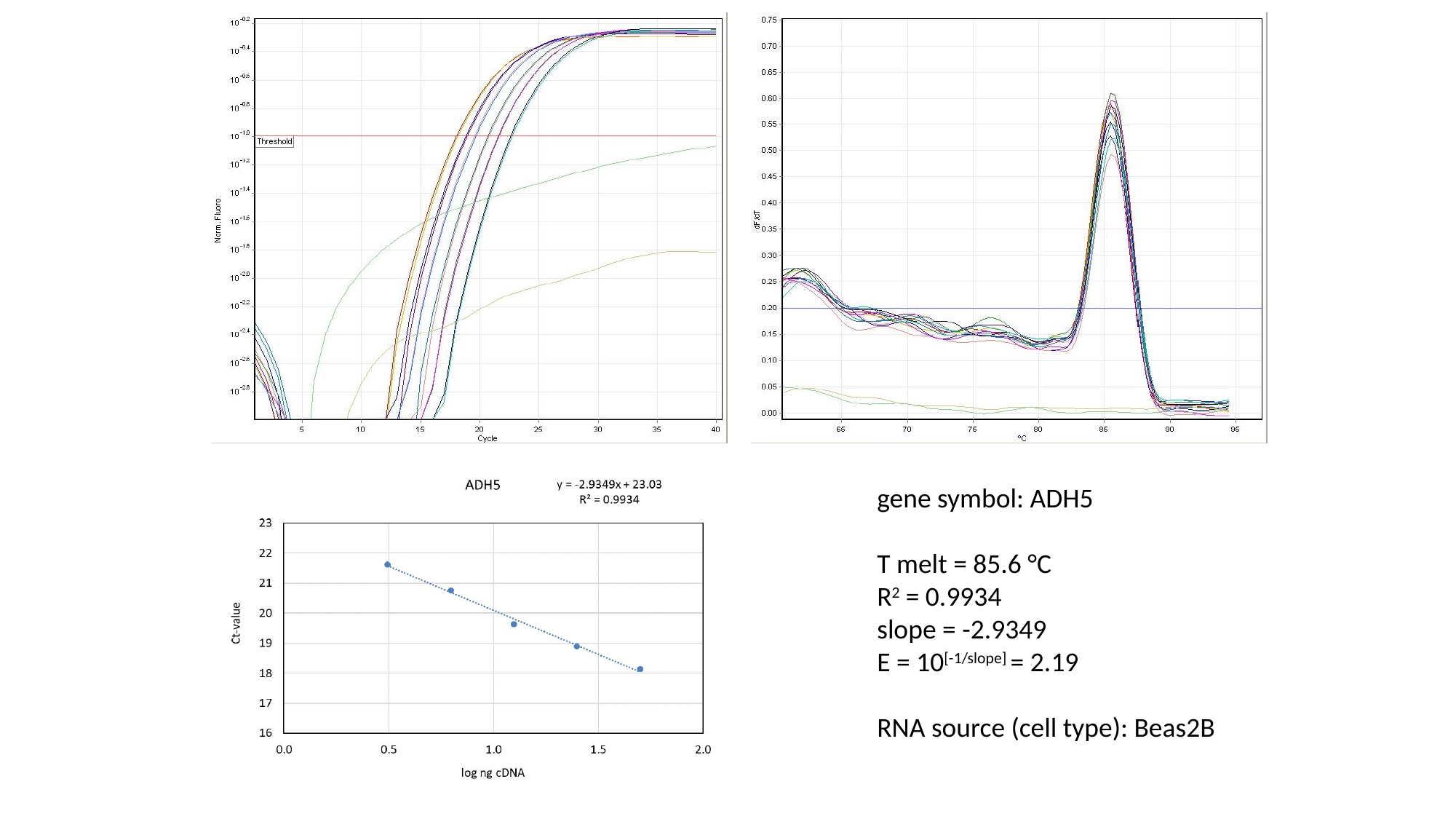

gene symbol: ADH5
T melt = 85.6 °C
R2 = 0.9934
slope = -2.9349
E = 10[-1/slope] = 2.19
RNA source (cell type): Beas2B

## Slide 4
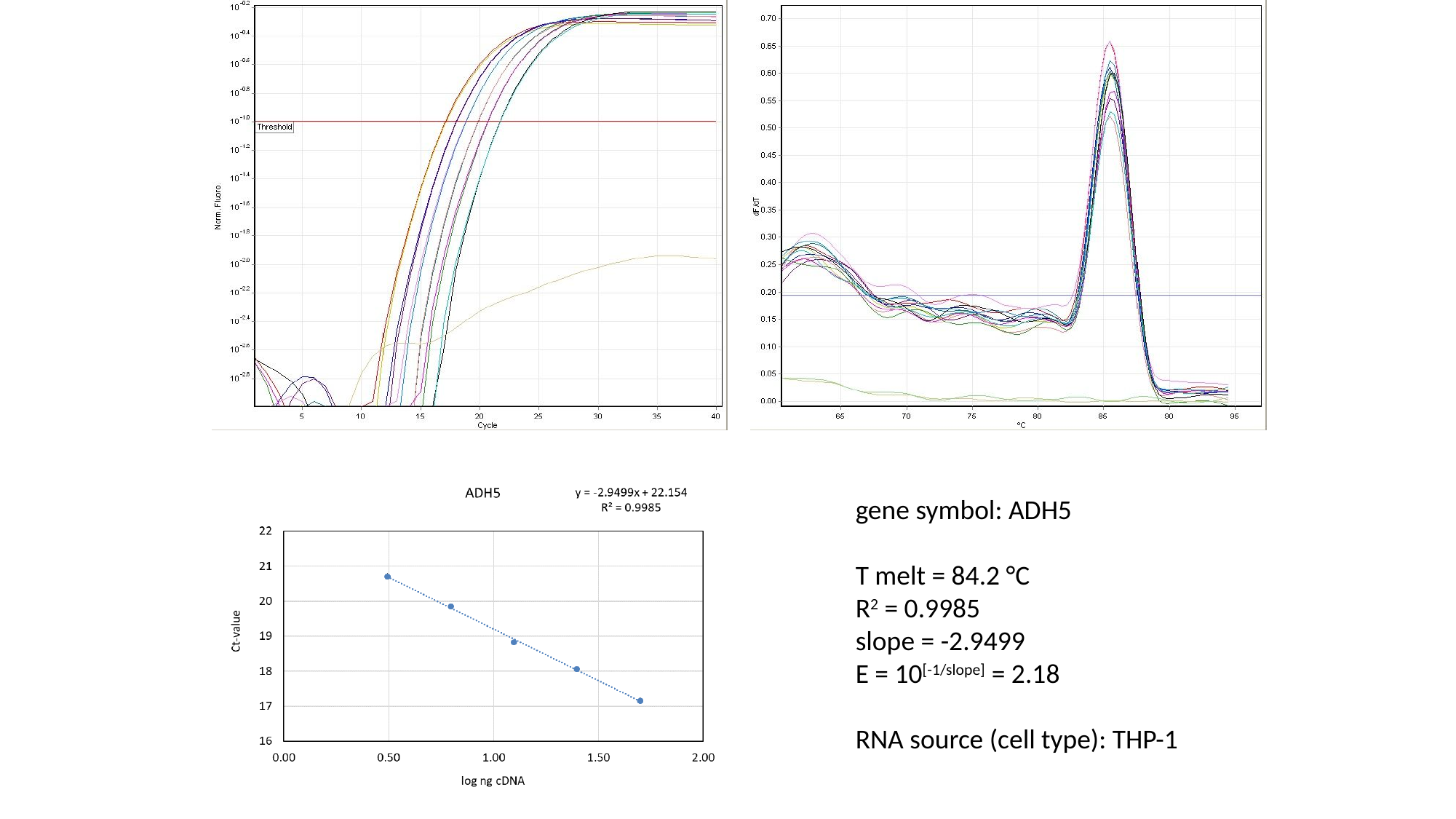

gene symbol: ADH5
T melt = 84.2 °C
R2 = 0.9985
slope = -2.9499
E = 10[-1/slope] = 2.18
RNA source (cell type): THP-1

## Slide 5
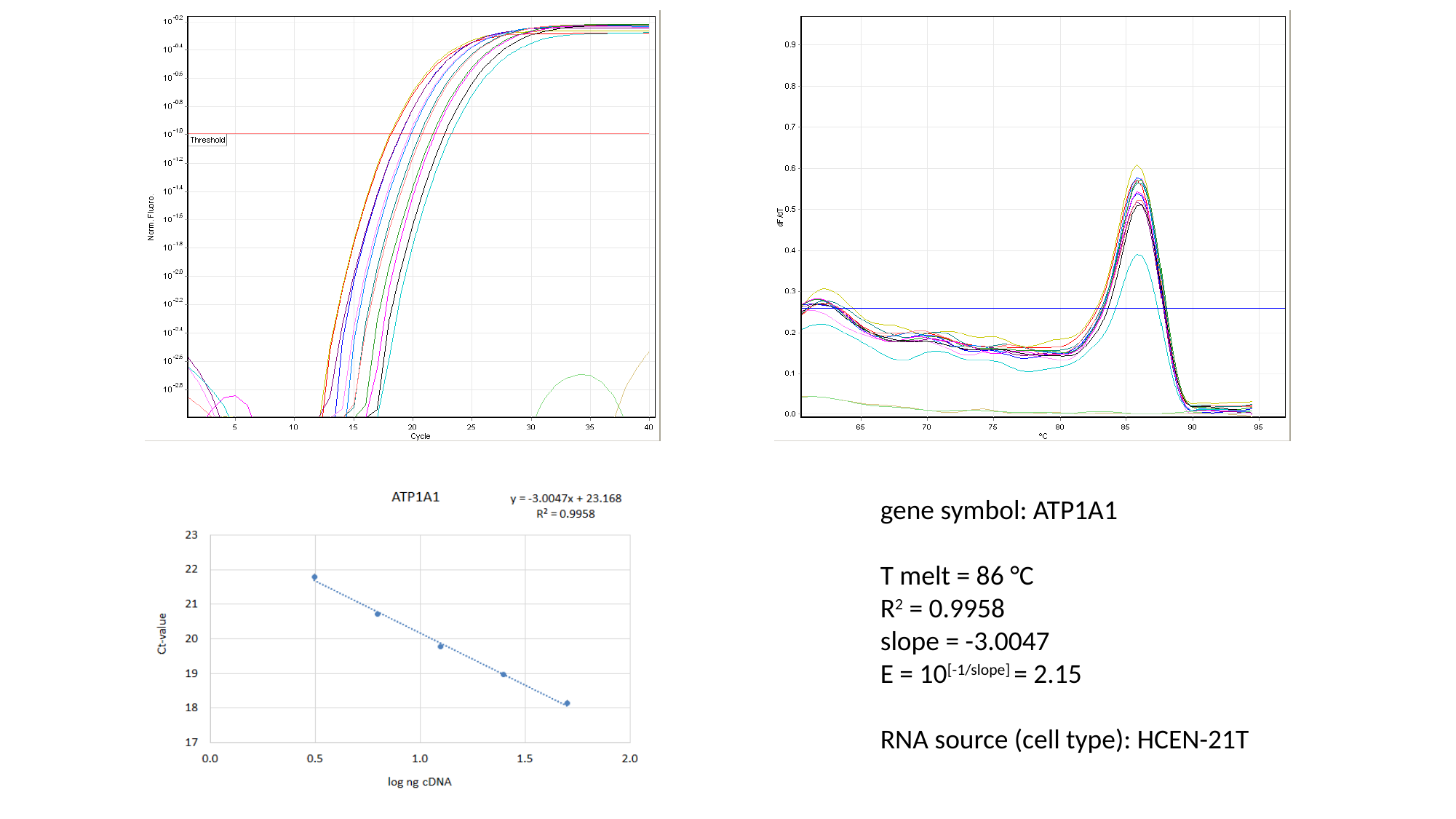

gene symbol: ATP1A1
T melt = 86 °C
R2 = 0.9958
slope = -3.0047
E = 10[-1/slope] = 2.15
RNA source (cell type): HCEN-21T

## Slide 6
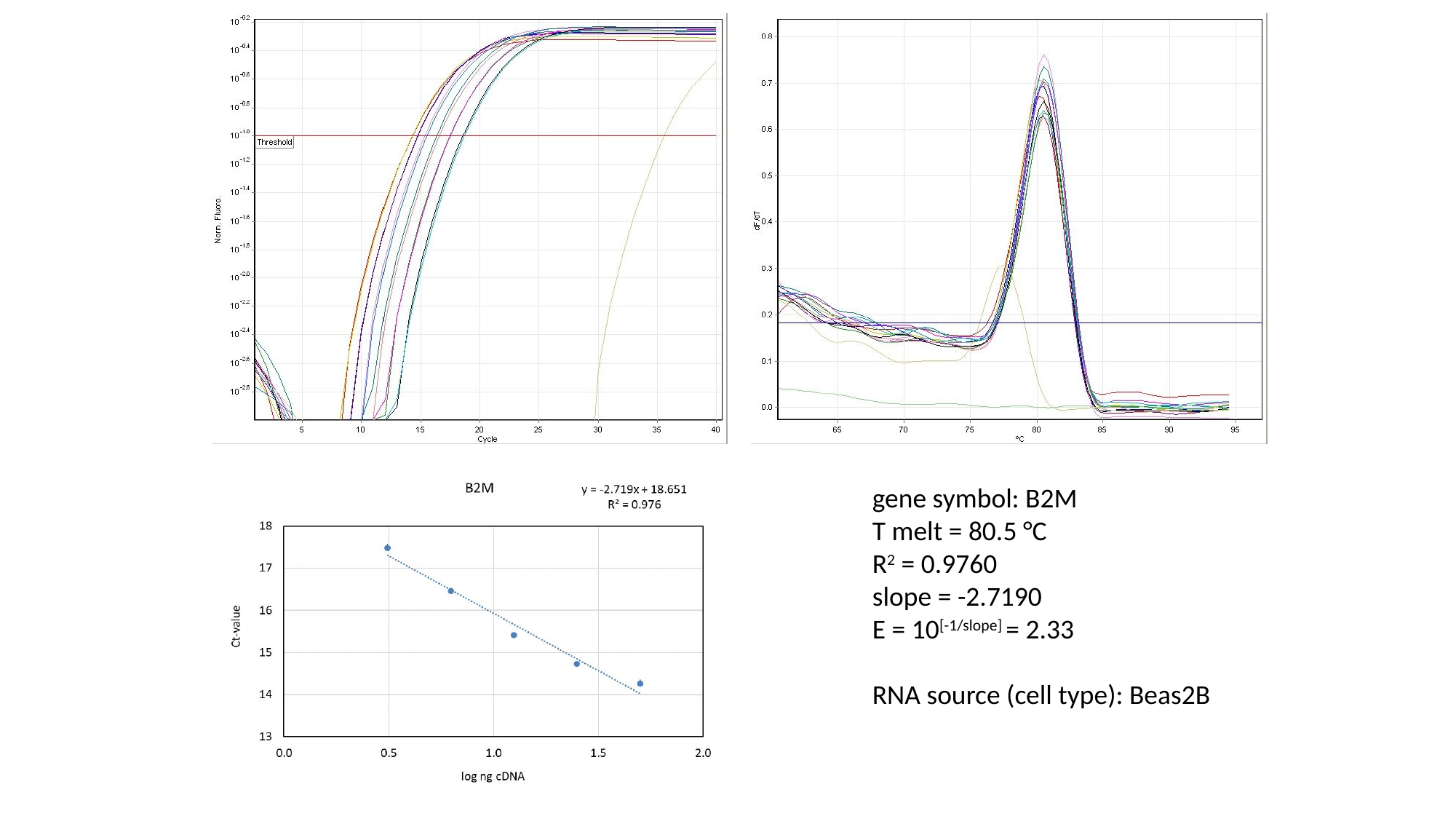

gene symbol: B2M
T melt = 80.5 °C
R2 = 0.9760
slope = -2.7190
E = 10[-1/slope] = 2.33
RNA source (cell type): Beas2B

## Slide 7
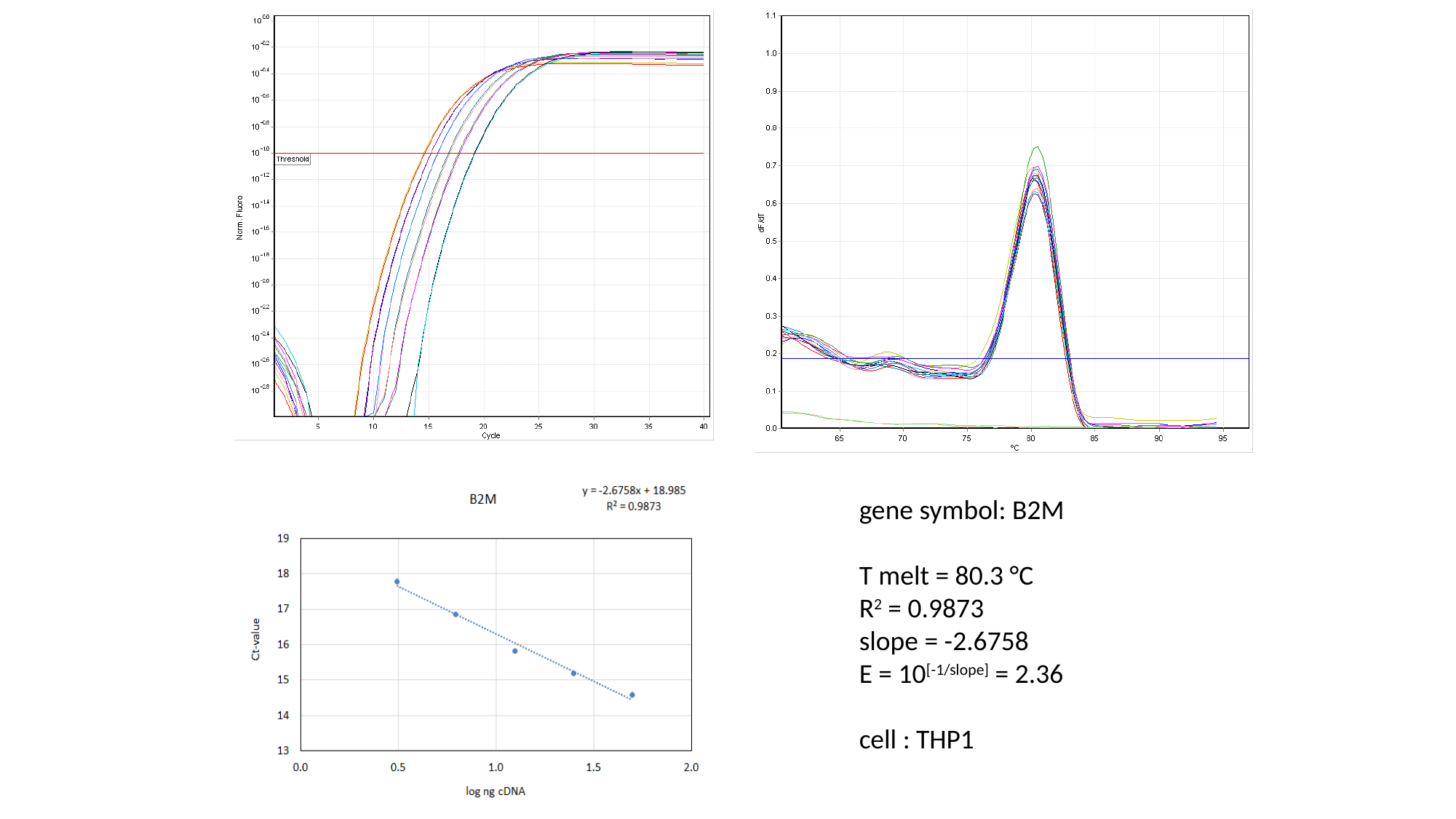

gene symbol: B2M
T melt = 80.3 °C
R2 = 0.9873
slope = -2.6758
E = 10[-1/slope] = 2.36
cell : THP1

## Slide 8
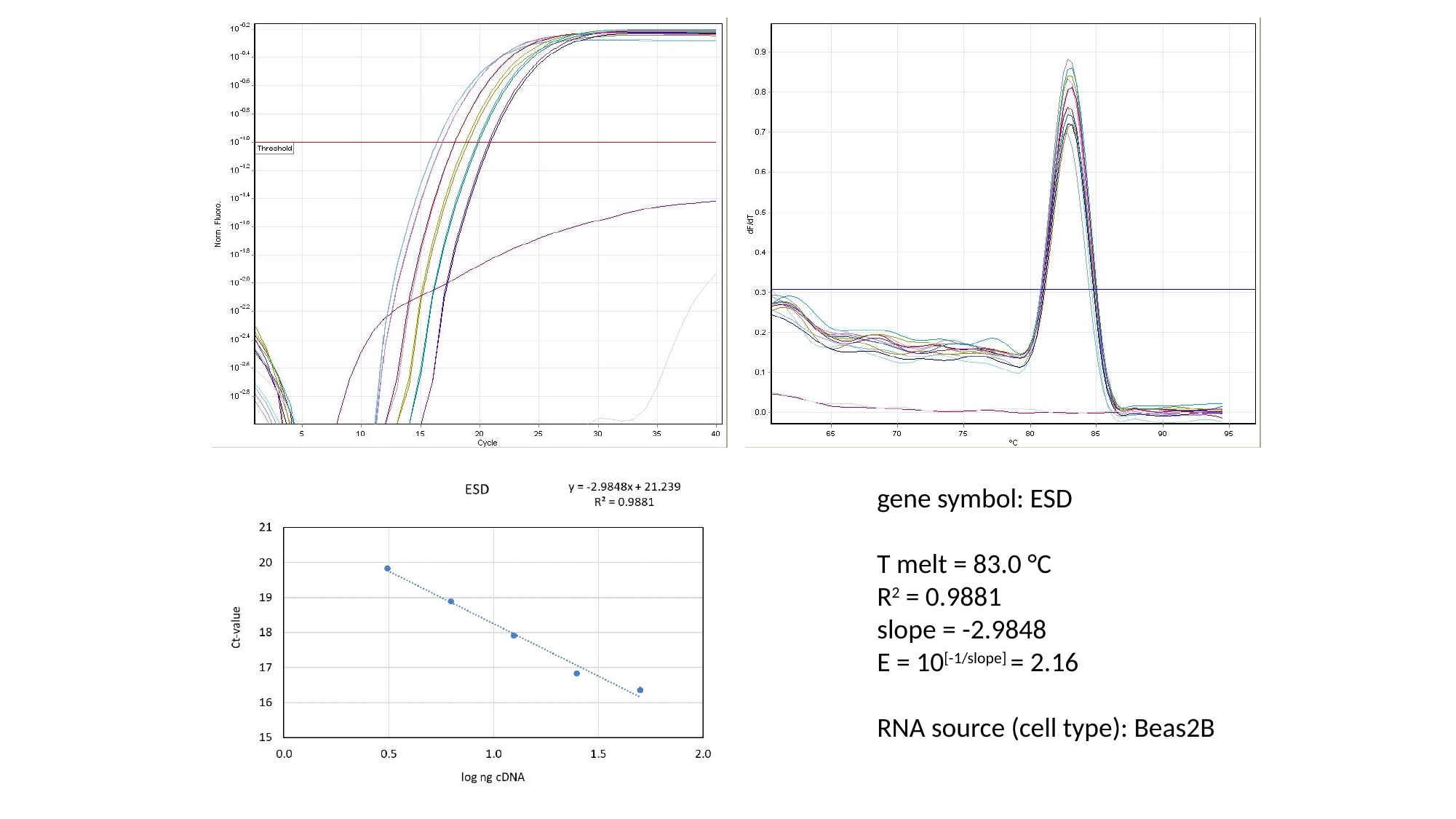

gene symbol: ESD
T melt = 83.0 °C
R2 = 0.9881
slope = -2.9848
E = 10[-1/slope] = 2.16
RNA source (cell type): Beas2B

## Slide 9
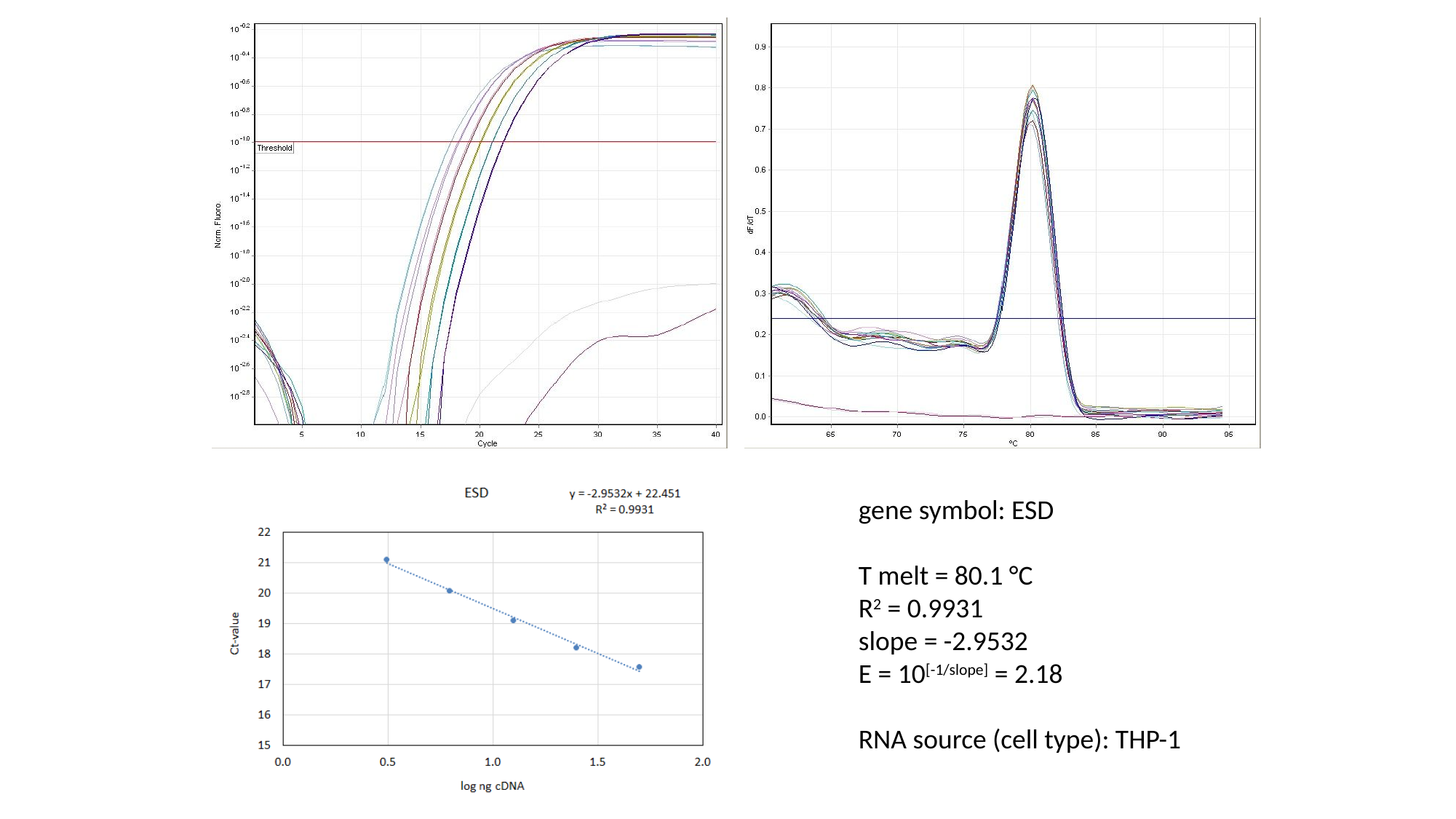

gene symbol: ESD
T melt = 80.1 °C
R2 = 0.9931
slope = -2.9532
E = 10[-1/slope] = 2.18
RNA source (cell type): THP-1

## Slide 10
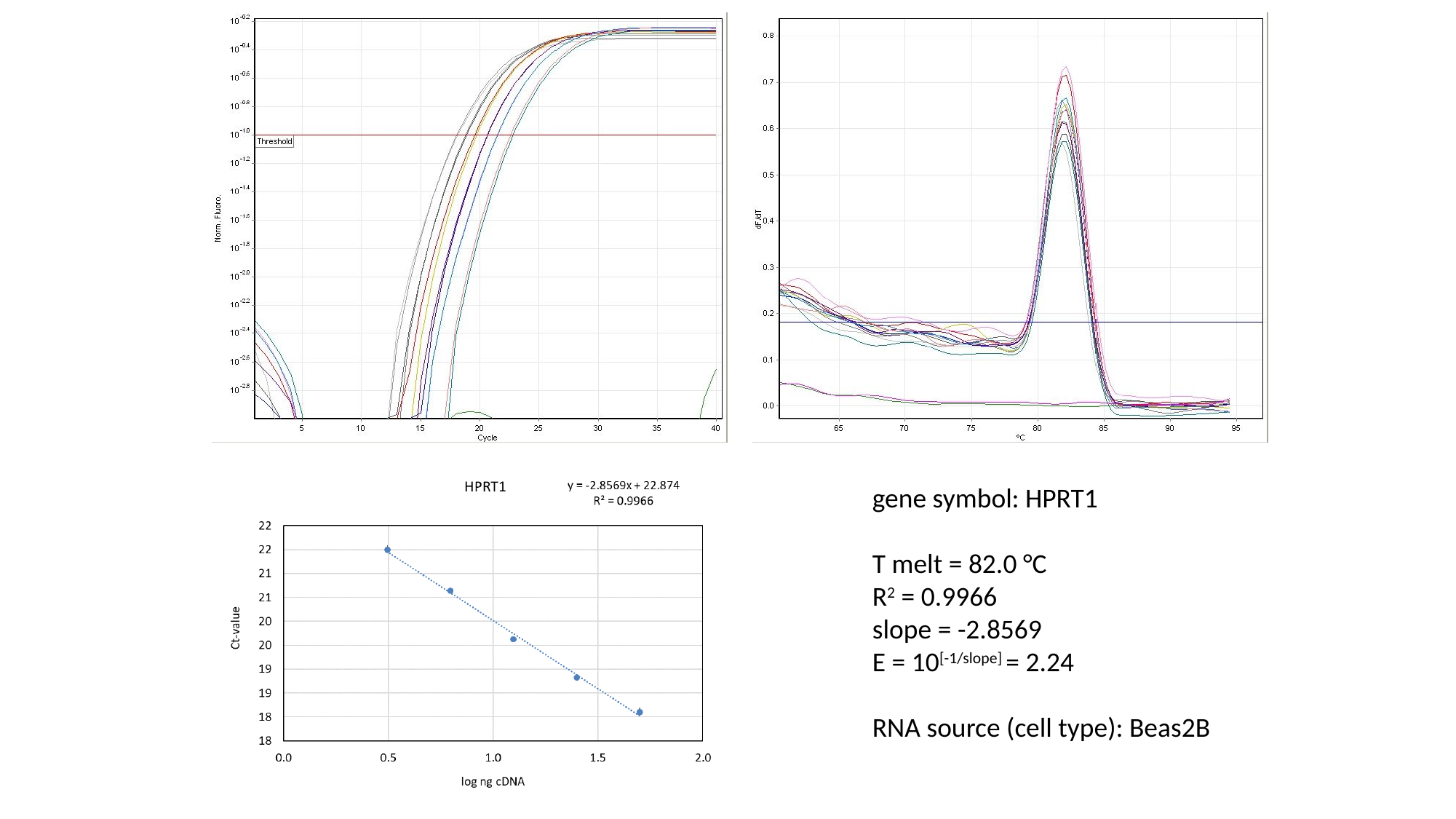

gene symbol: HPRT1
T melt = 82.0 °C
R2 = 0.9966
slope = -2.8569
E = 10[-1/slope] = 2.24
RNA source (cell type): Beas2B

## Slide 11
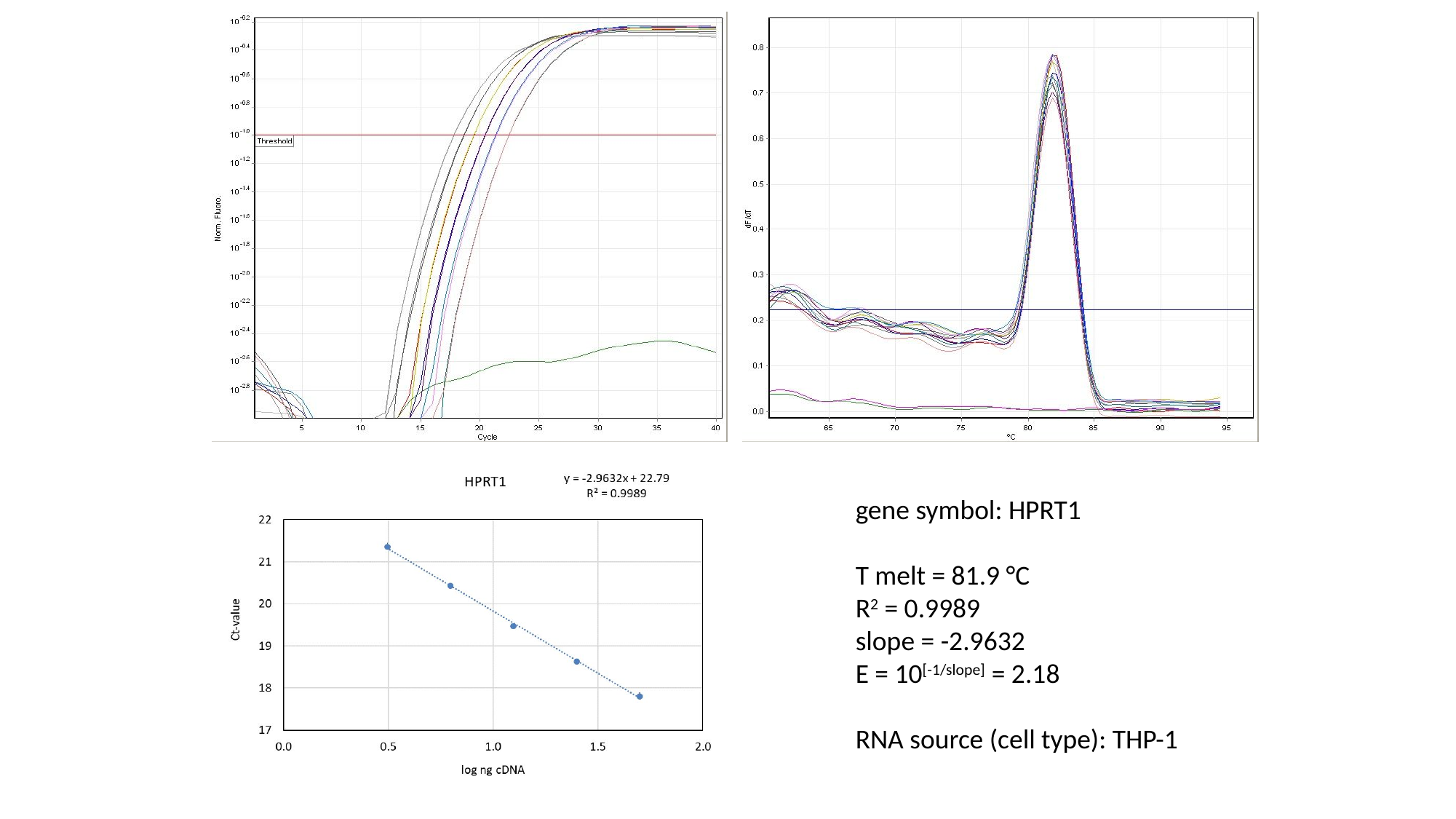

gene symbol: HPRT1
T melt = 81.9 °C
R2 = 0.9989
slope = -2.9632
E = 10[-1/slope] = 2.18
RNA source (cell type): THP-1

## Slide 12
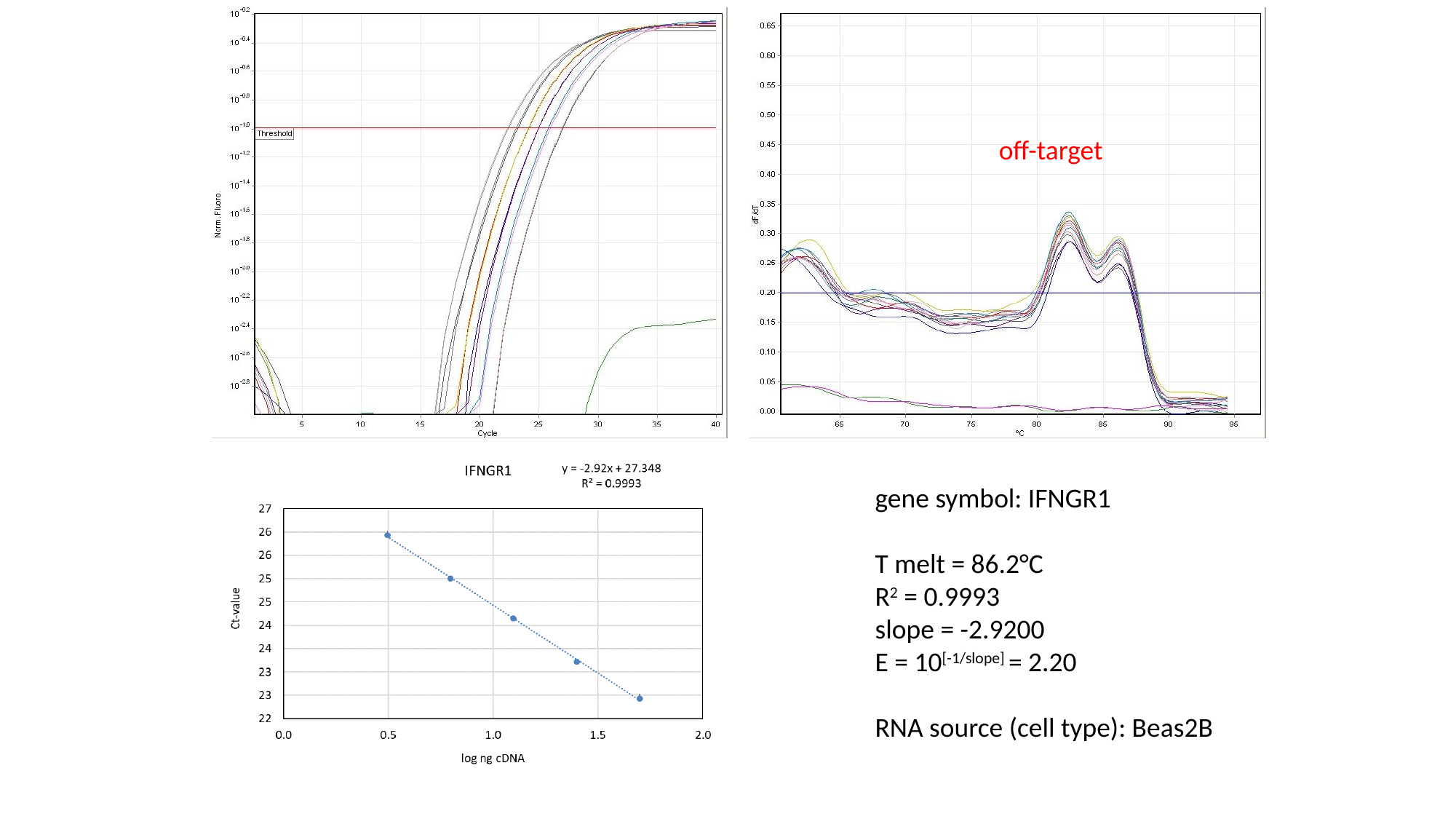

off-target
gene symbol: IFNGR1
T melt = 86.2°C
R2 = 0.9993
slope = -2.9200
E = 10[-1/slope] = 2.20
RNA source (cell type): Beas2B

## Slide 13
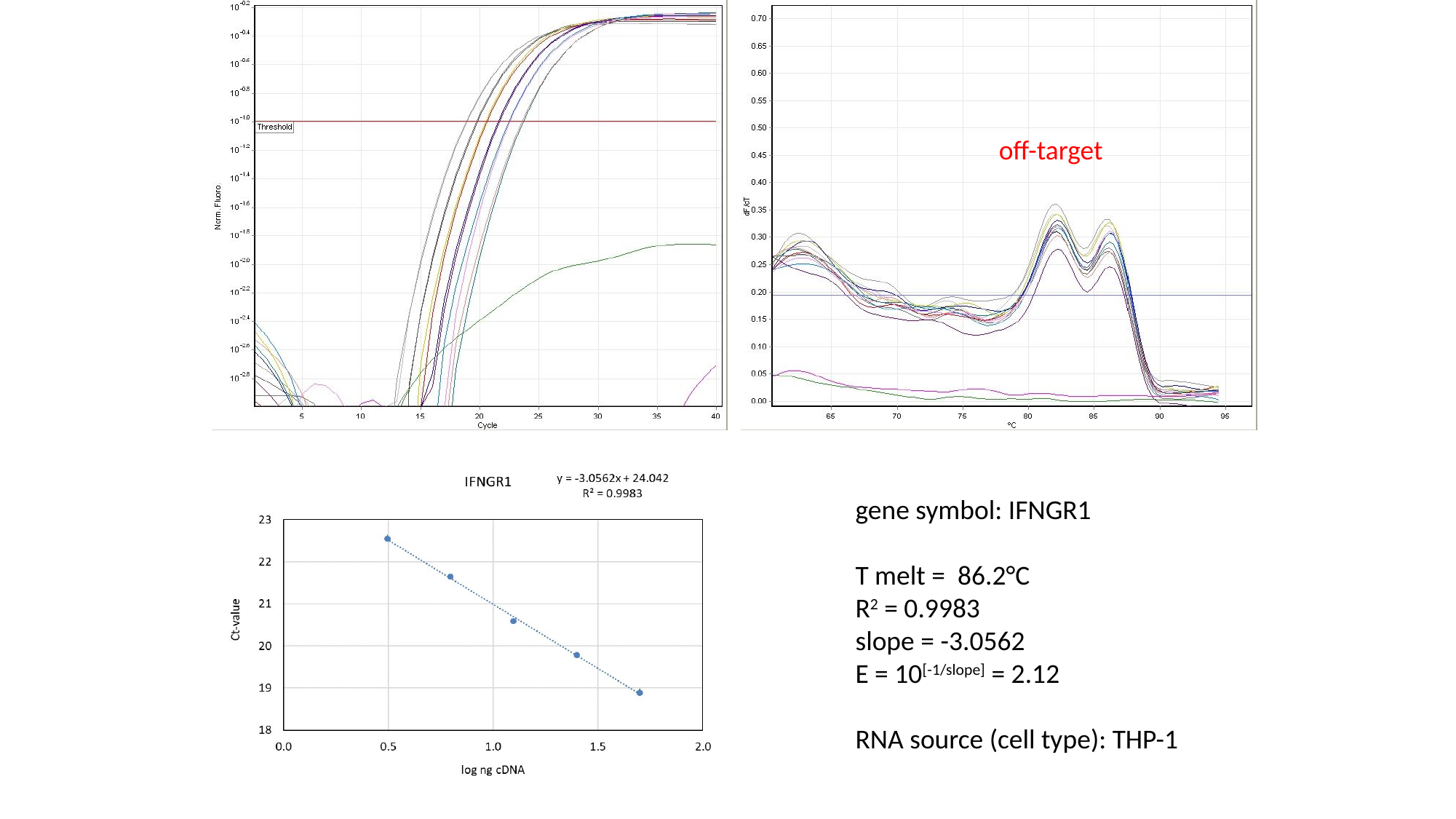

off-target
gene symbol: IFNGR1
T melt = 86.2°C
R2 = 0.9983
slope = -3.0562
E = 10[-1/slope] = 2.12
RNA source (cell type): THP-1

## Slide 14
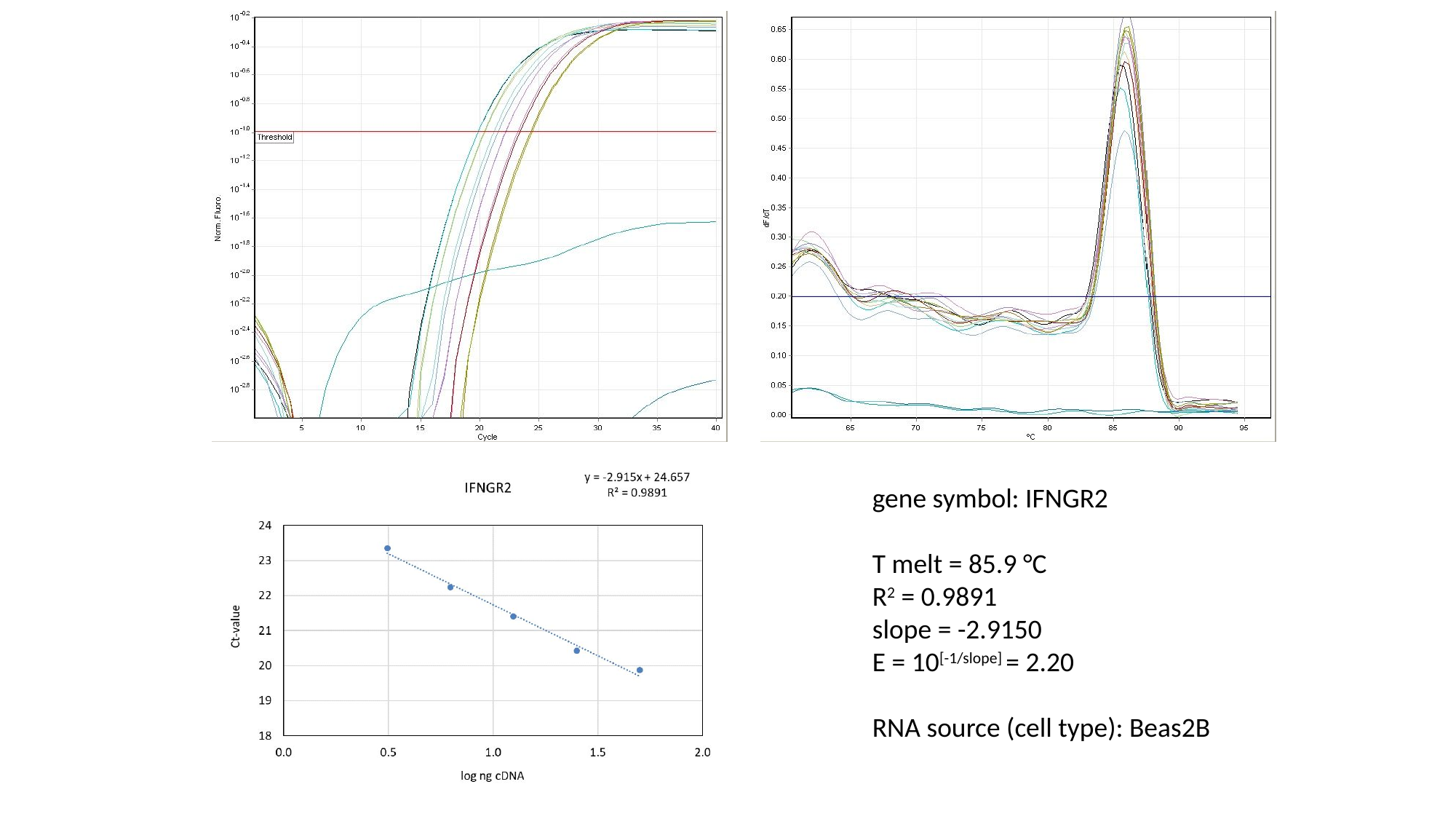

gene symbol: IFNGR2
T melt = 85.9 °C
R2 = 0.9891
slope = -2.9150
E = 10[-1/slope] = 2.20
RNA source (cell type): Beas2B

## Slide 15
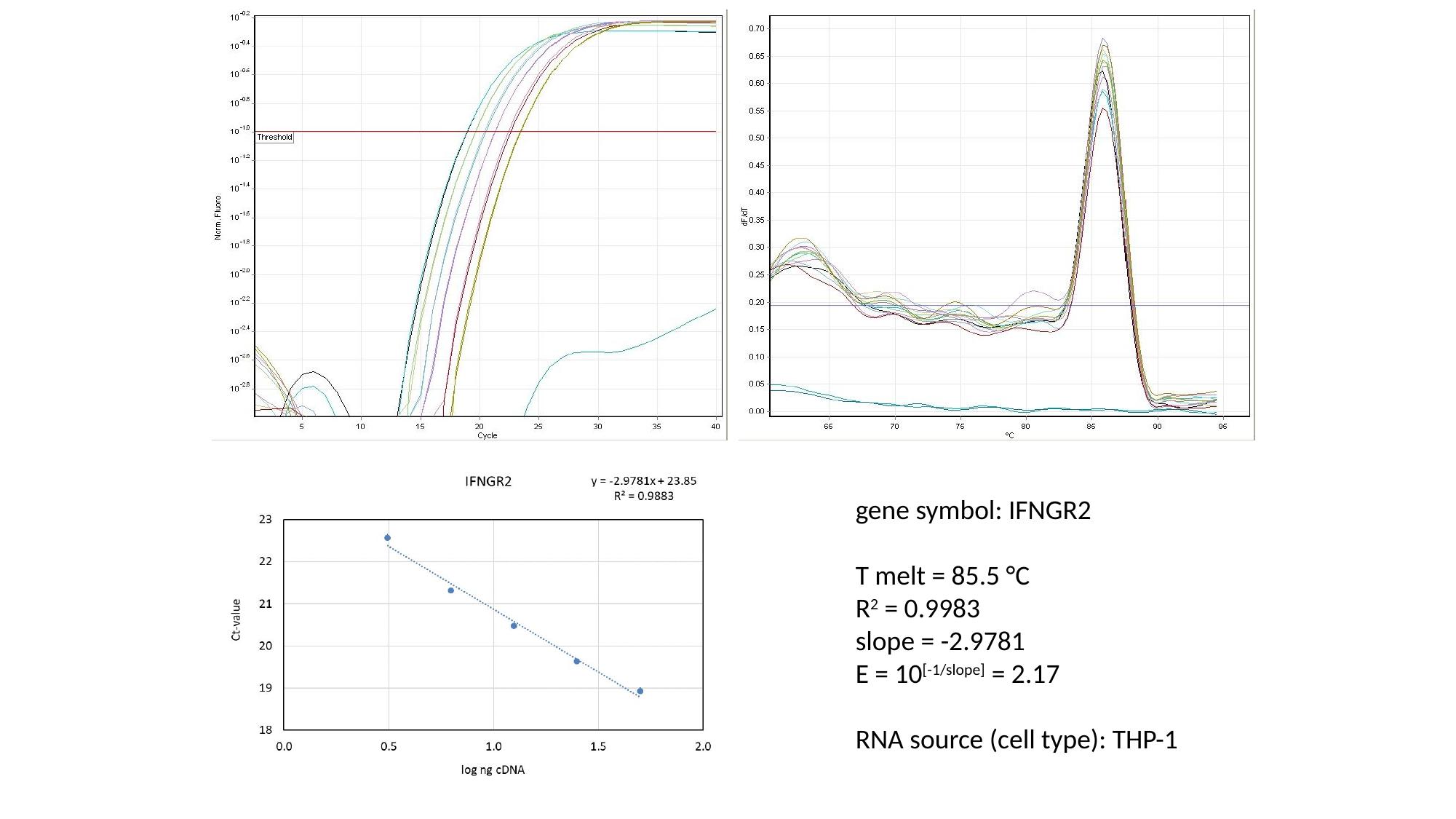

gene symbol: IFNGR2
T melt = 85.5 °C
R2 = 0.9983
slope = -2.9781
E = 10[-1/slope] = 2.17
RNA source (cell type): THP-1

## Slide 16
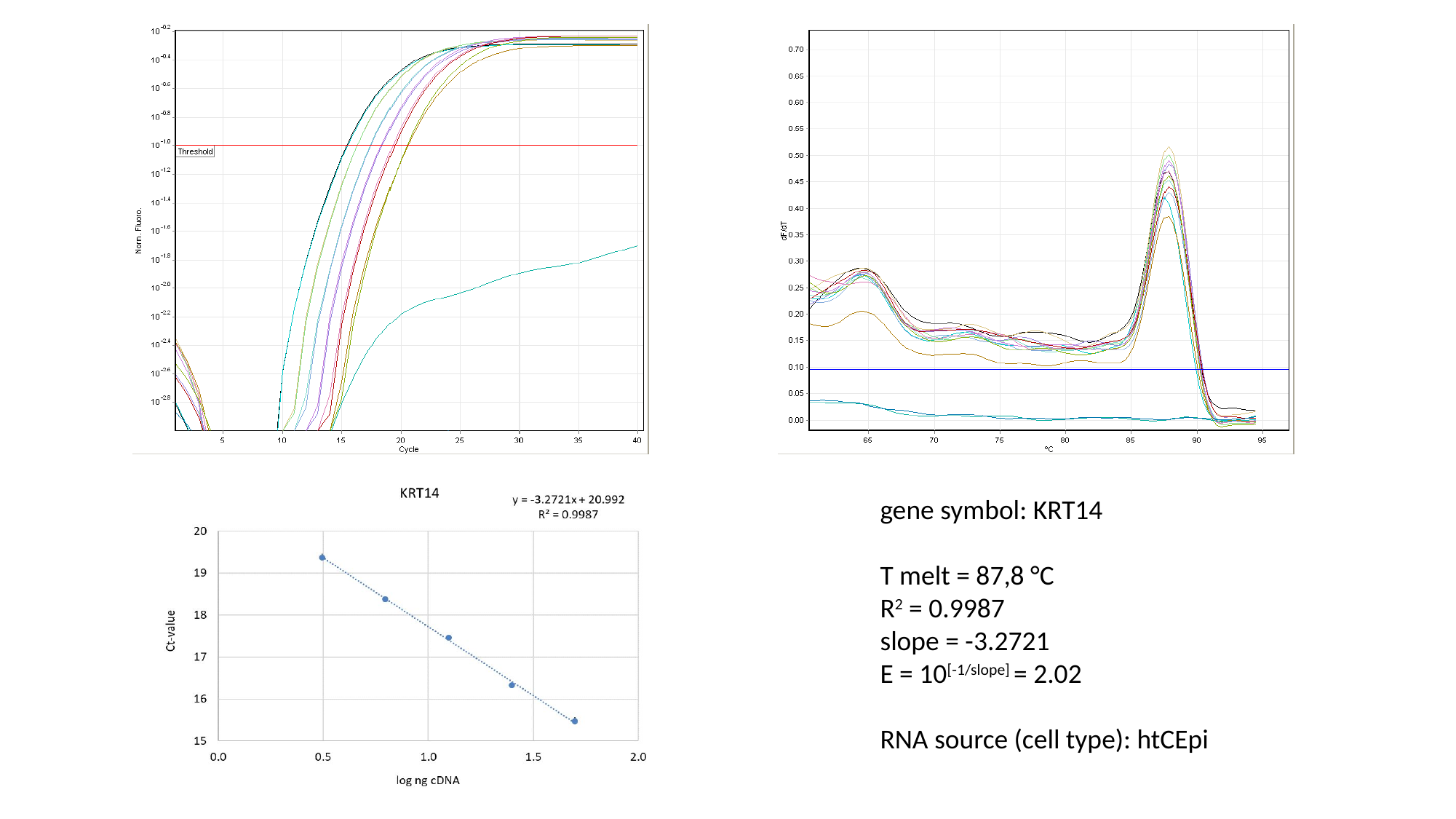

gene symbol: KRT14
T melt = 87,8 °C
R2 = 0.9987
slope = -3.2721
E = 10[-1/slope] = 2.02
RNA source (cell type): htCEpi

## Slide 17
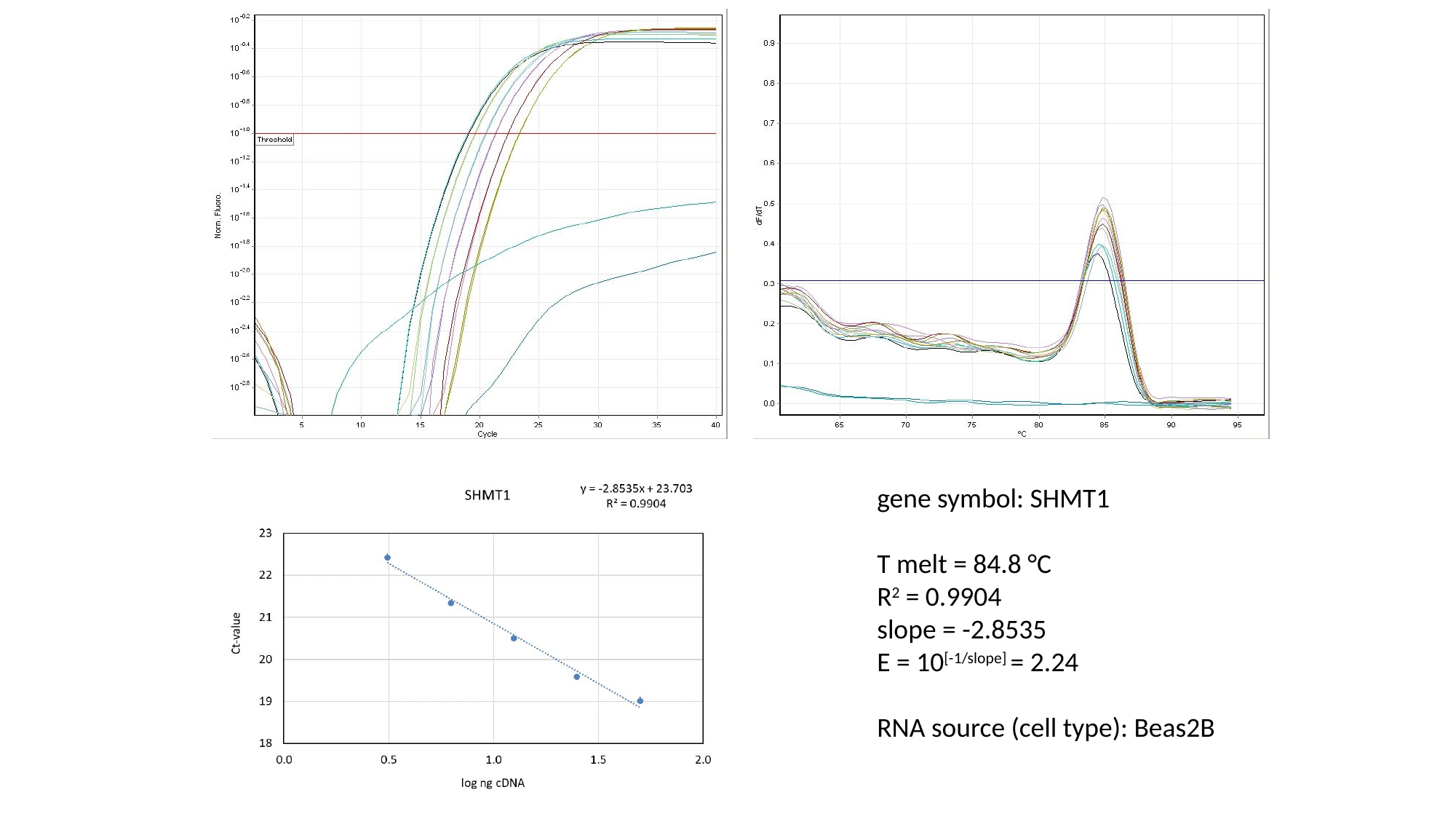

gene symbol: SHMT1
T melt = 84.8 °C
R2 = 0.9904
slope = -2.8535
E = 10[-1/slope] = 2.24
RNA source (cell type): Beas2B

## Slide 18
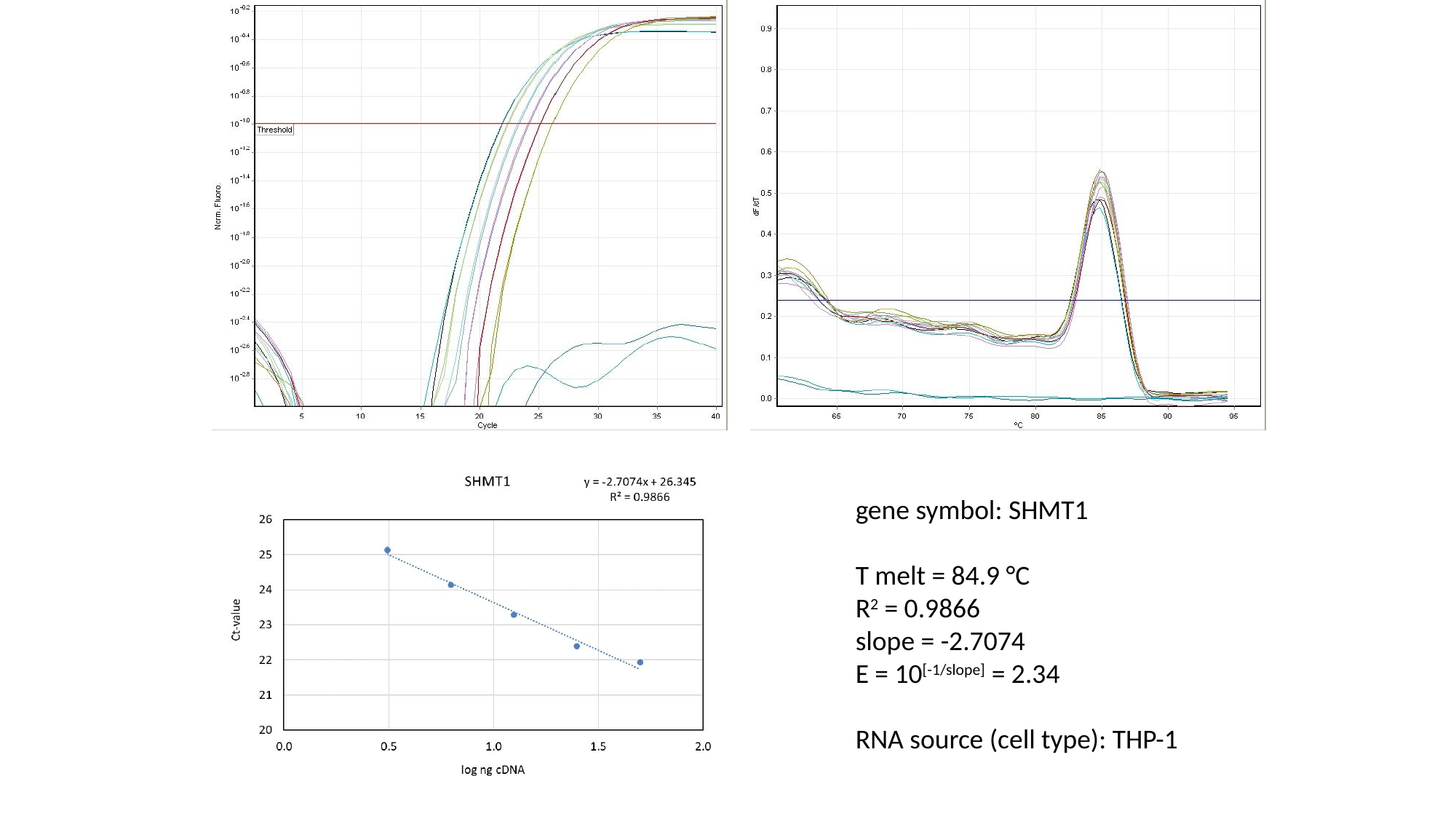

gene symbol: SHMT1
T melt = 84.9 °C
R2 = 0.9866
slope = -2.7074
E = 10[-1/slope] = 2.34
RNA source (cell type): THP-1

## Slide 19
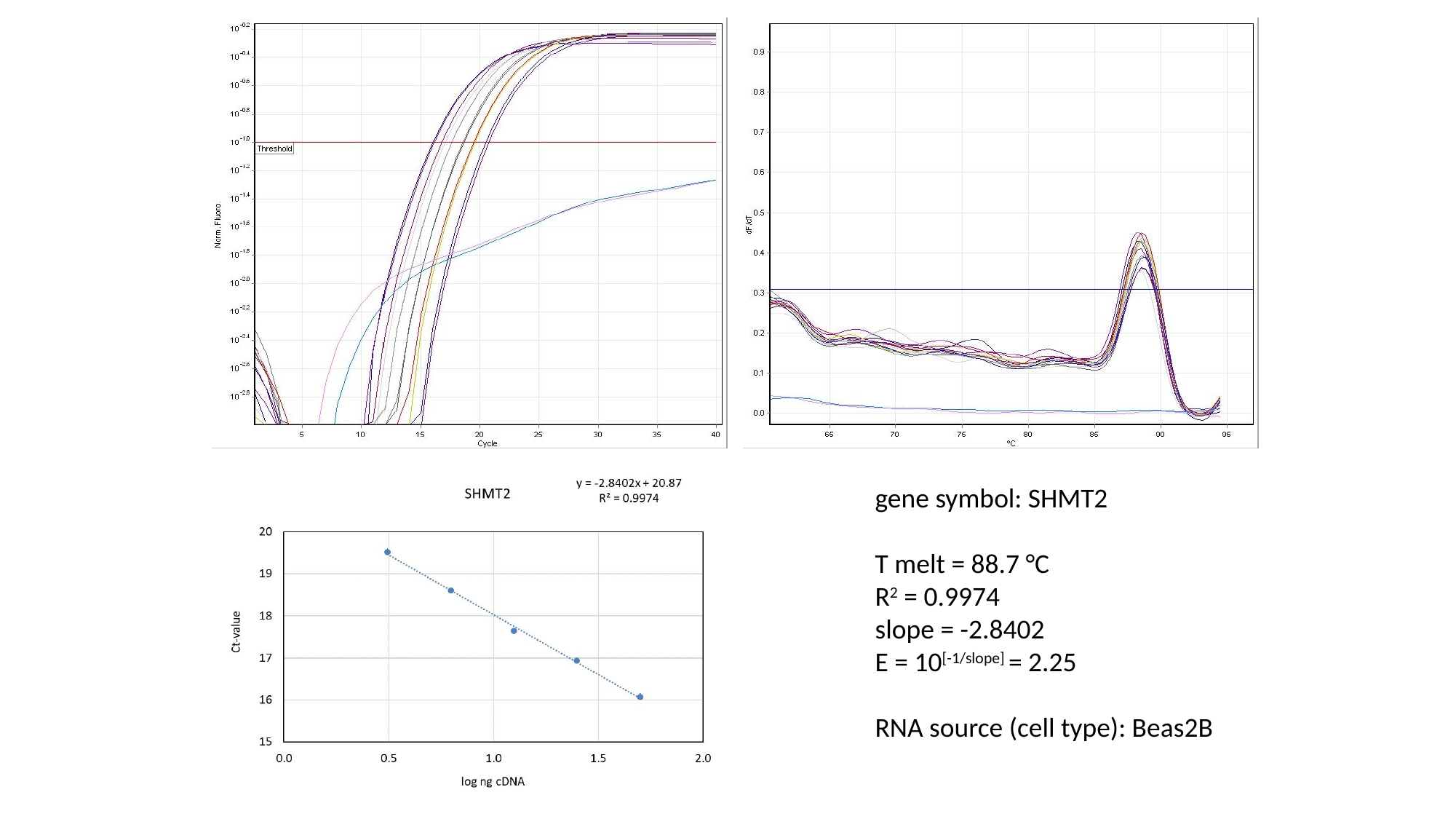

gene symbol: SHMT2
T melt = 88.7 °C
R2 = 0.9974
slope = -2.8402
E = 10[-1/slope] = 2.25
RNA source (cell type): Beas2B

## Slide 20
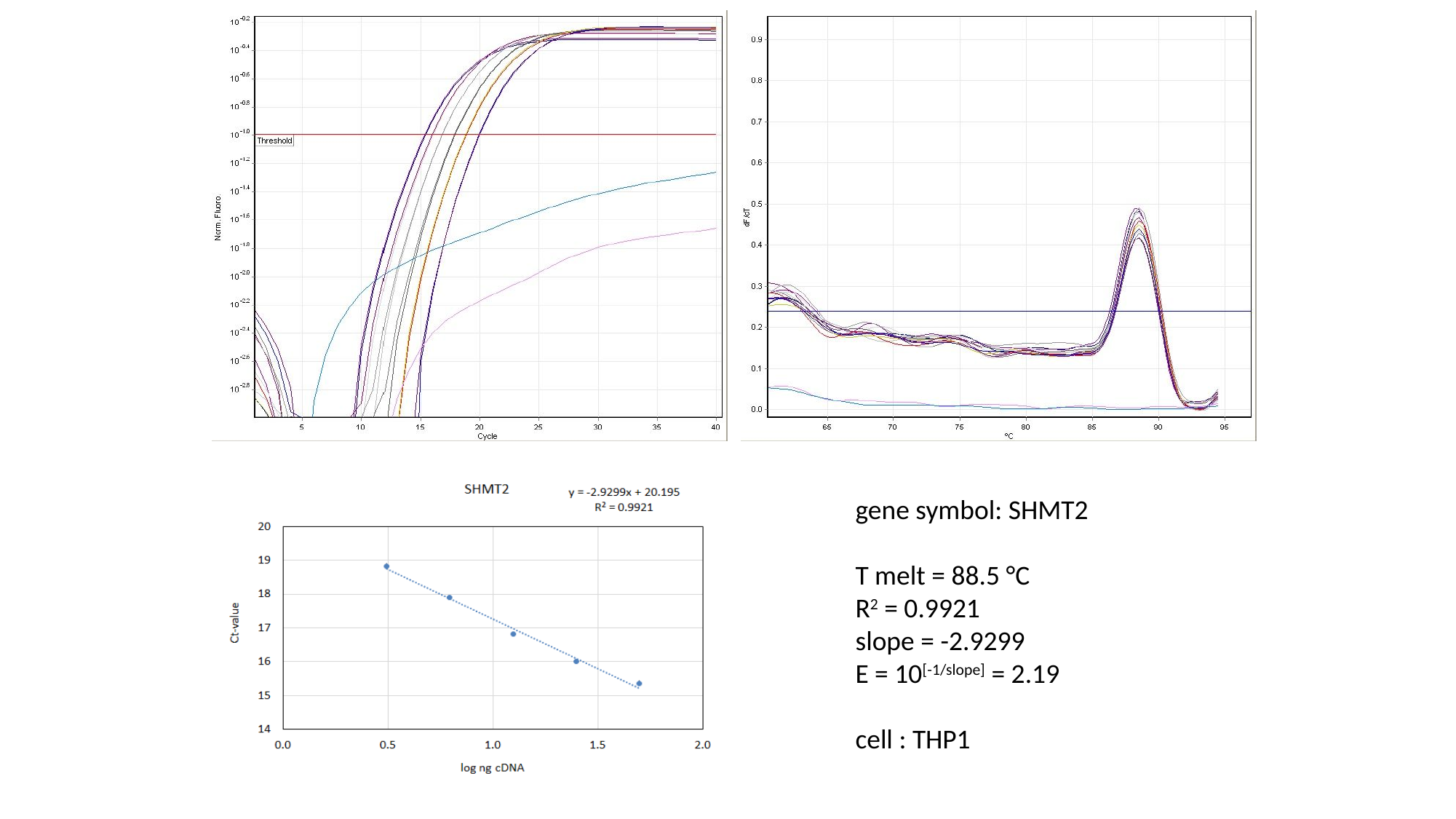

gene symbol: SHMT2
T melt = 88.5 °C
R2 = 0.9921
slope = -2.9299
E = 10[-1/slope] = 2.19
cell : THP1

## Slide 21
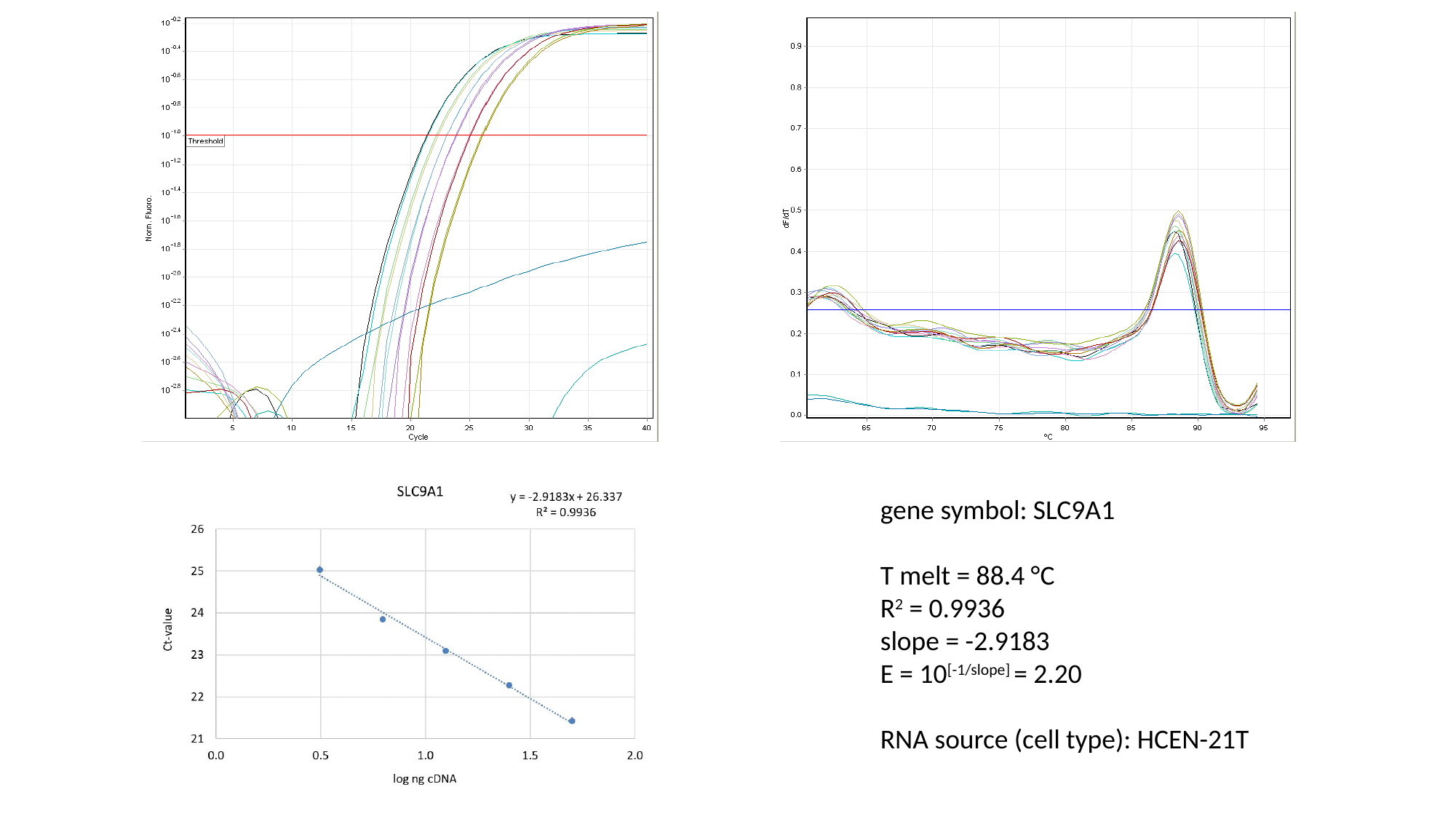

gene symbol: SLC9A1
T melt = 88.4 °C
R2 = 0.9936
slope = -2.9183
E = 10[-1/slope] = 2.20
RNA source (cell type): HCEN-21T

## Slide 22
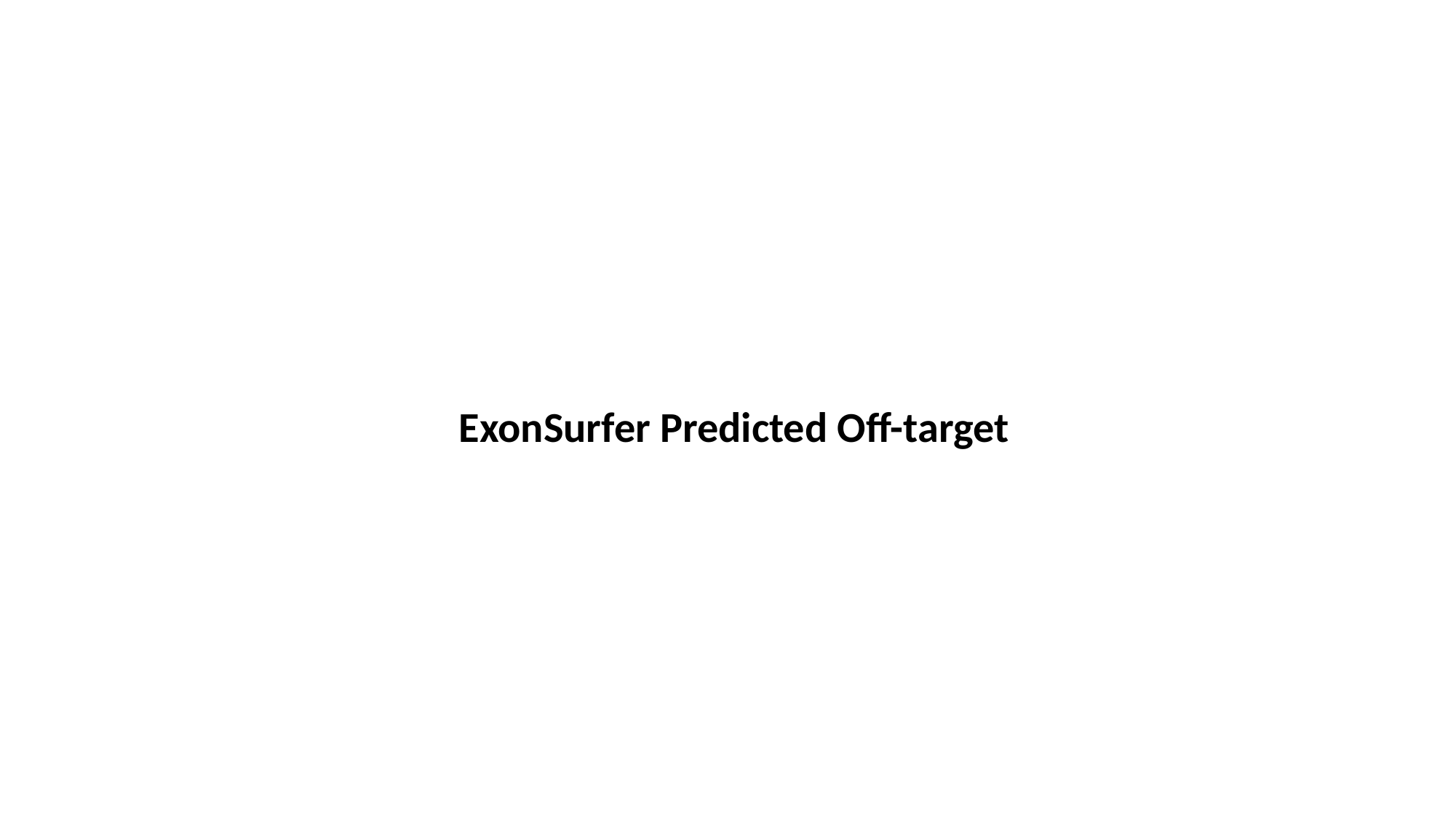

ExonSurfer Predicted Off-target

## Slide 23
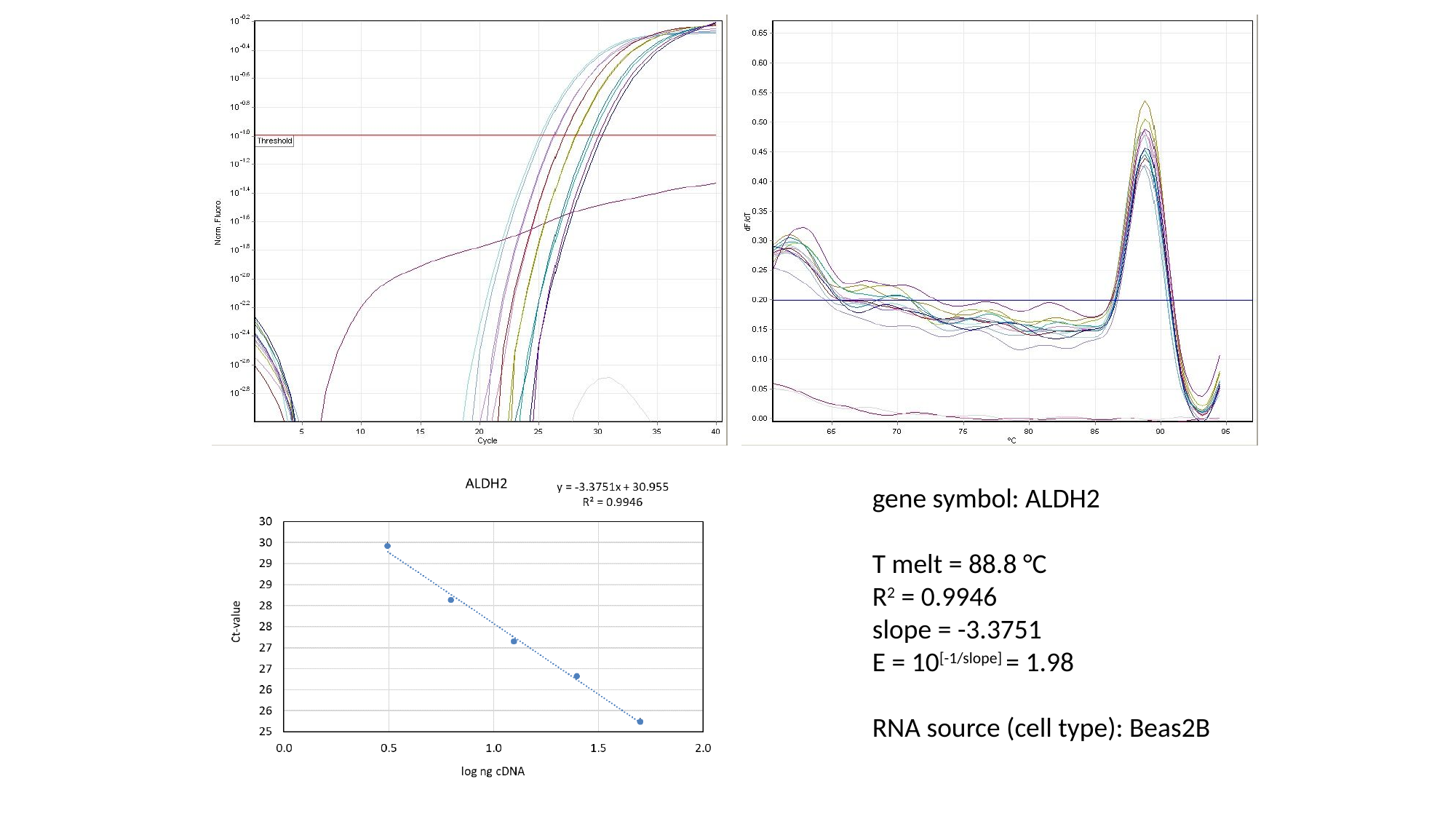

gene symbol: ALDH2
T melt = 88.8 °C
R2 = 0.9946
slope = -3.3751
E = 10[-1/slope] = 1.98
RNA source (cell type): Beas2B

## Slide 24
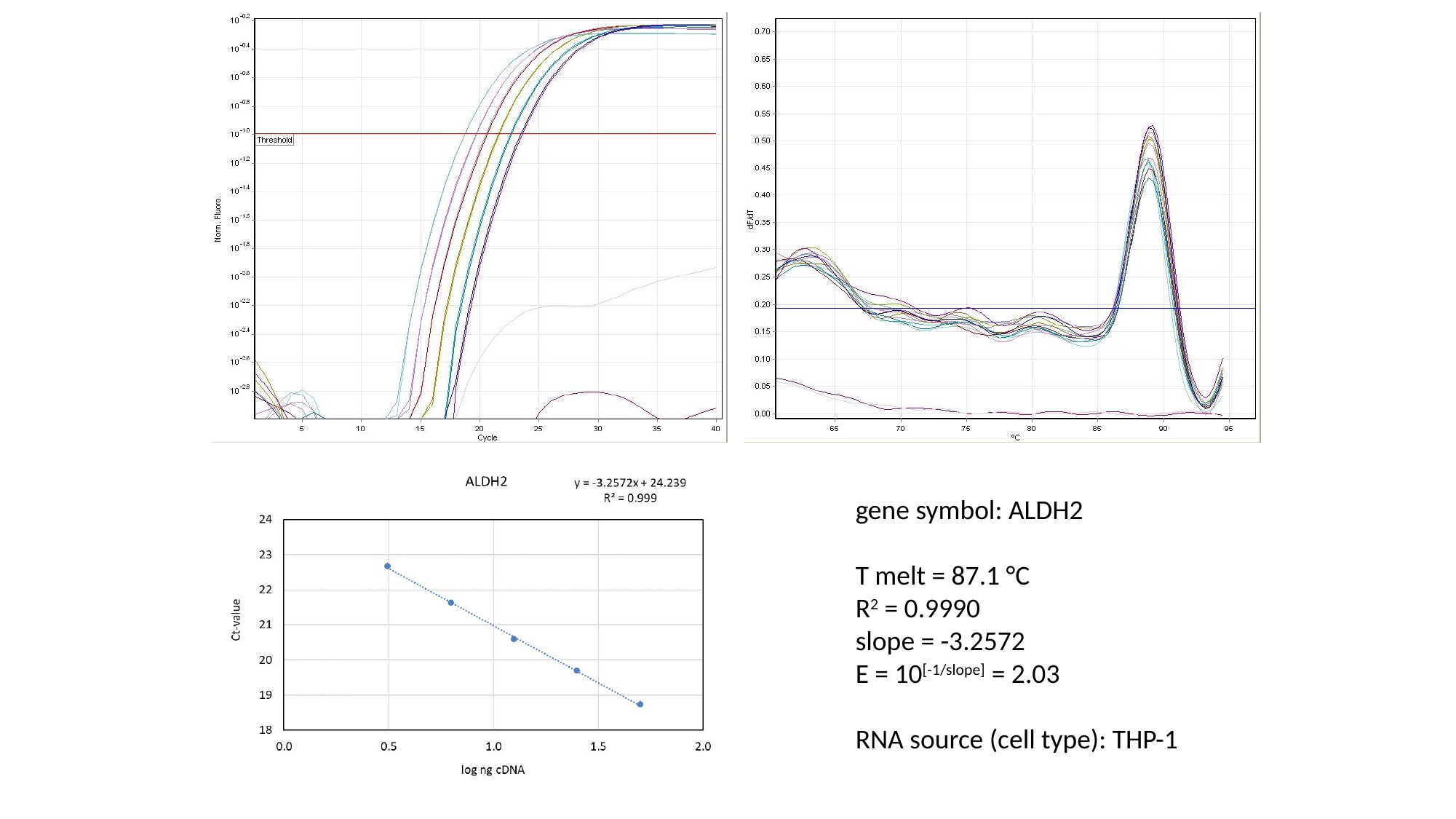

gene symbol: ALDH2
T melt = 87.1 °C
R2 = 0.9990
slope = -3.2572
E = 10[-1/slope] = 2.03
RNA source (cell type): THP-1

## Slide 25
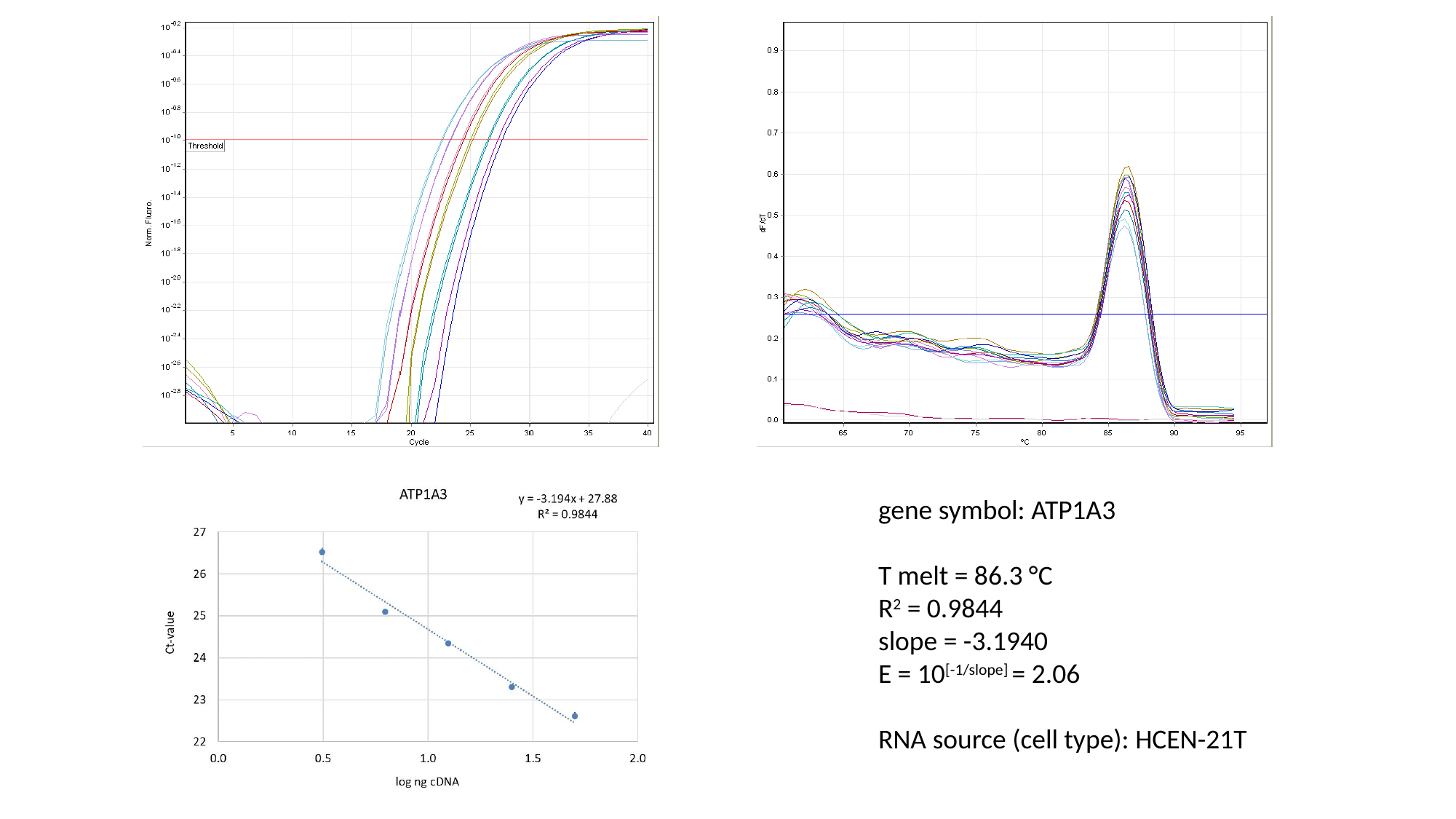

gene symbol: ATP1A3
T melt = 86.3 °C
R2 = 0.9844
slope = -3.1940
E = 10[-1/slope] = 2.06
RNA source (cell type): HCEN-21T

## Slide 26
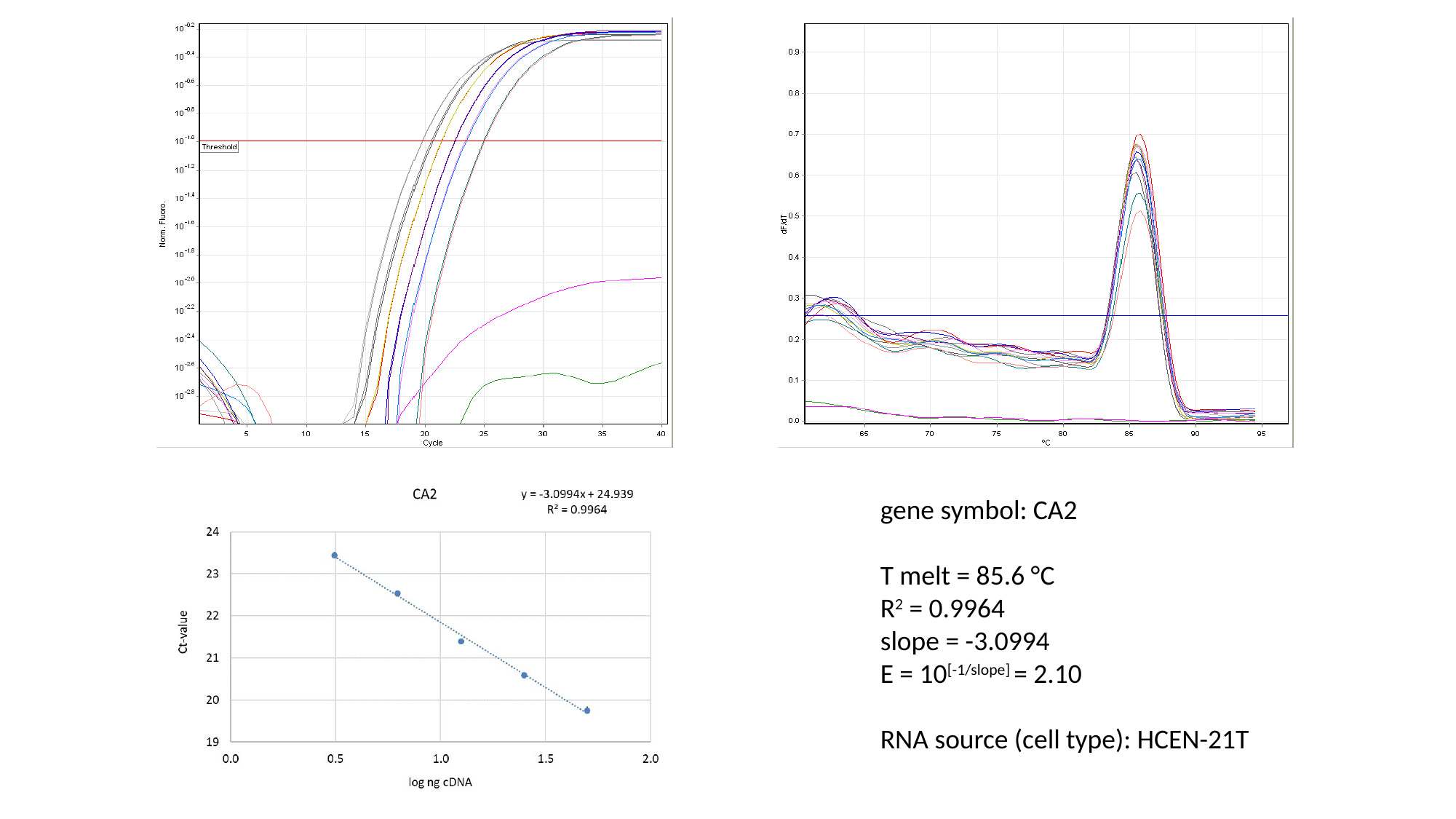

gene symbol: CA2
T melt = 85.6 °C
R2 = 0.9964
slope = -3.0994
E = 10[-1/slope] = 2.10
RNA source (cell type): HCEN-21T

## Slide 27
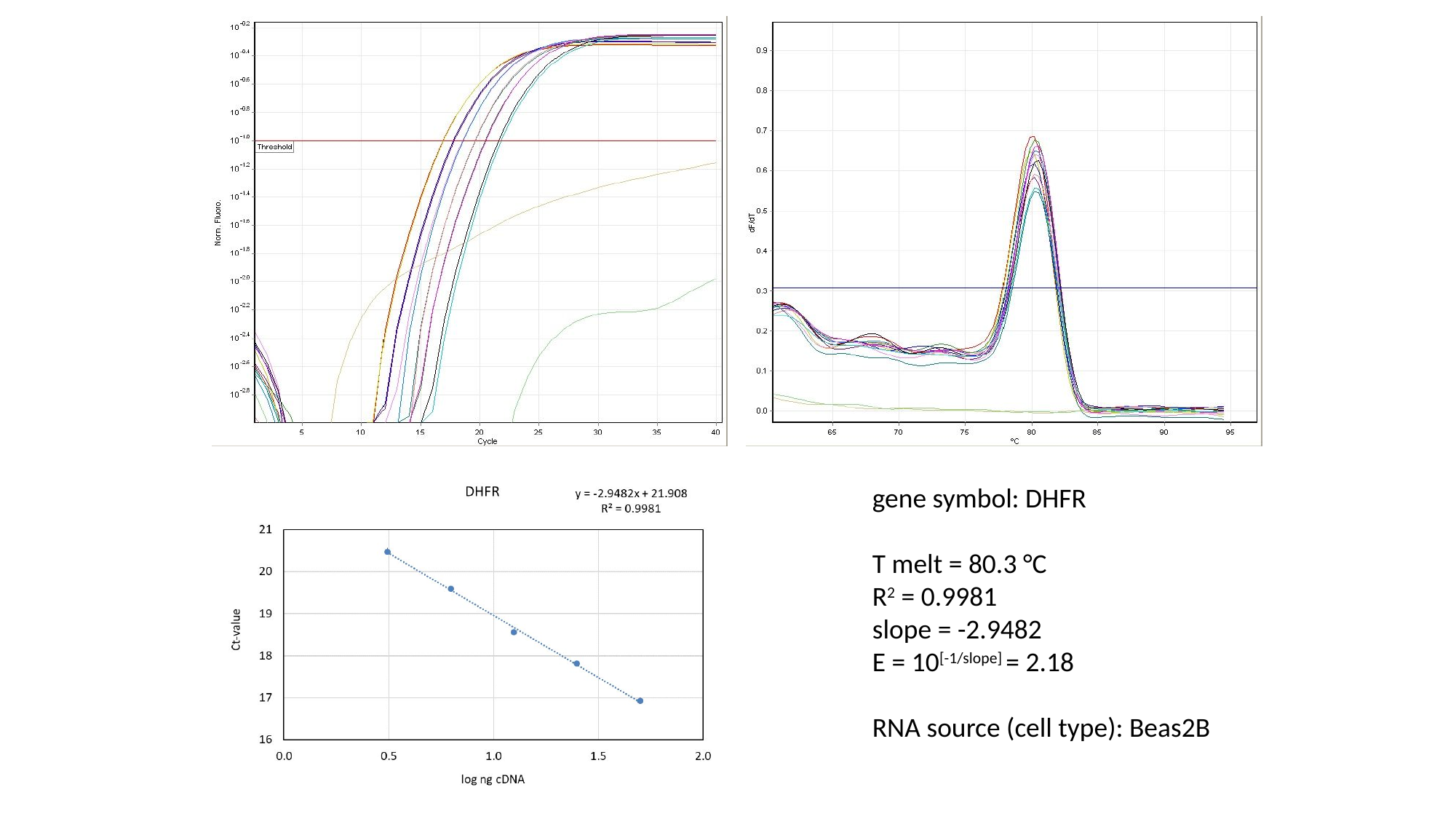

gene symbol: DHFR
T melt = 80.3 °C
R2 = 0.9981
slope = -2.9482
E = 10[-1/slope] = 2.18
RNA source (cell type): Beas2B

## Slide 28
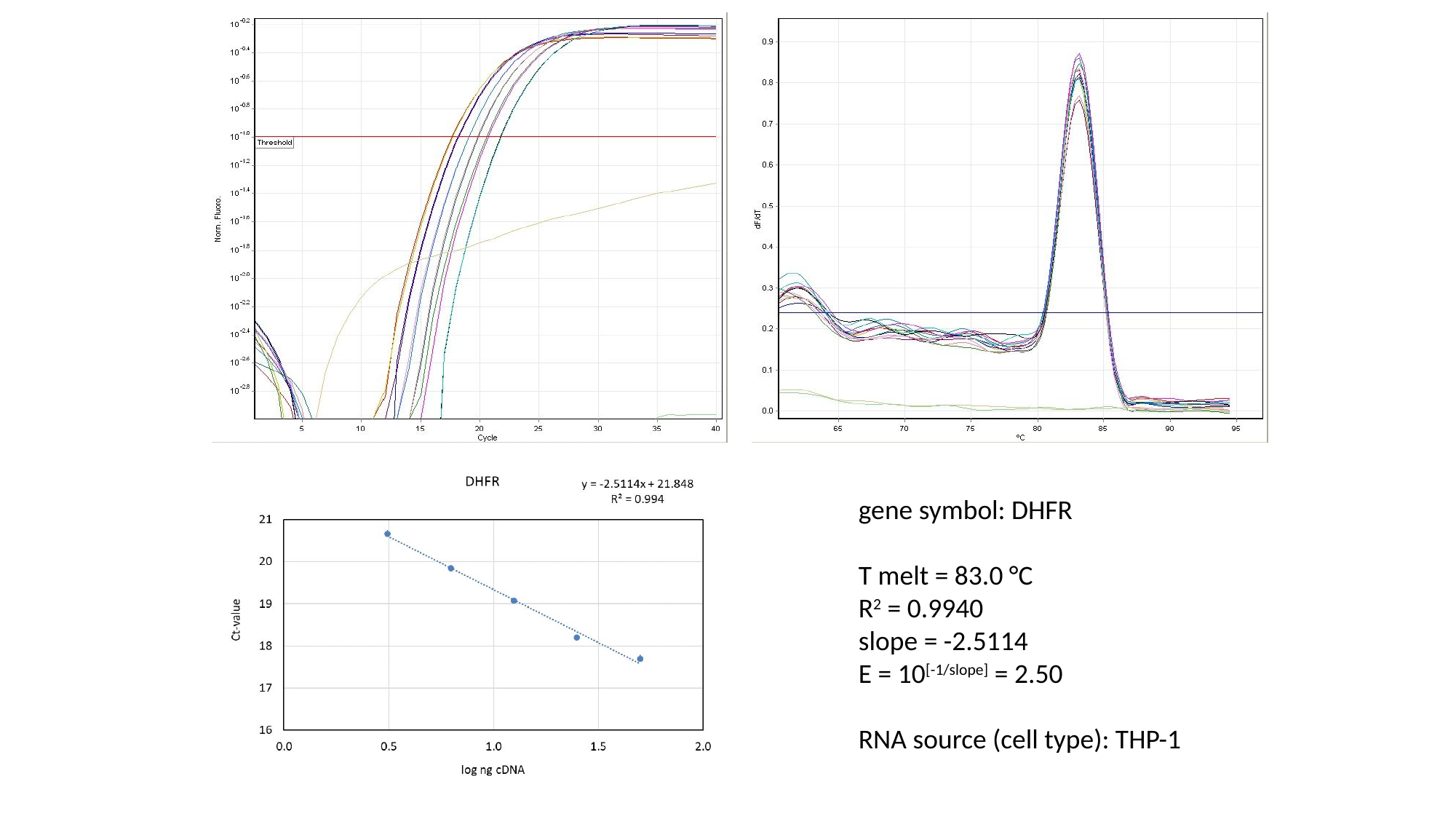

gene symbol: DHFR
T melt = 83.0 °C
R2 = 0.9940
slope = -2.5114
E = 10[-1/slope] = 2.50
RNA source (cell type): THP-1

## Slide 29
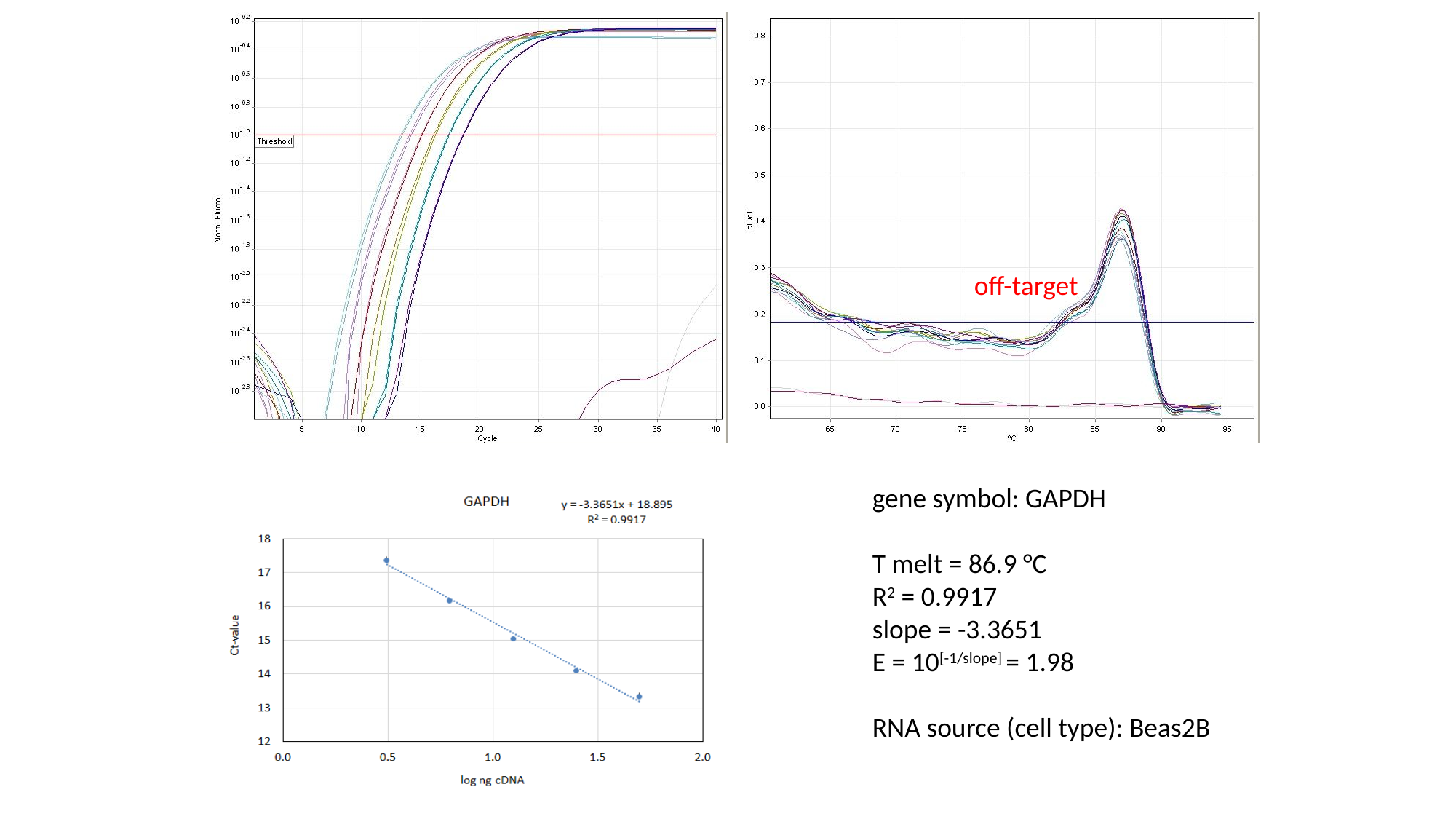

off-target
gene symbol: GAPDH
T melt = 86.9 °C
R2 = 0.9917
slope = -3.3651
E = 10[-1/slope] = 1.98
RNA source (cell type): Beas2B

## Slide 30
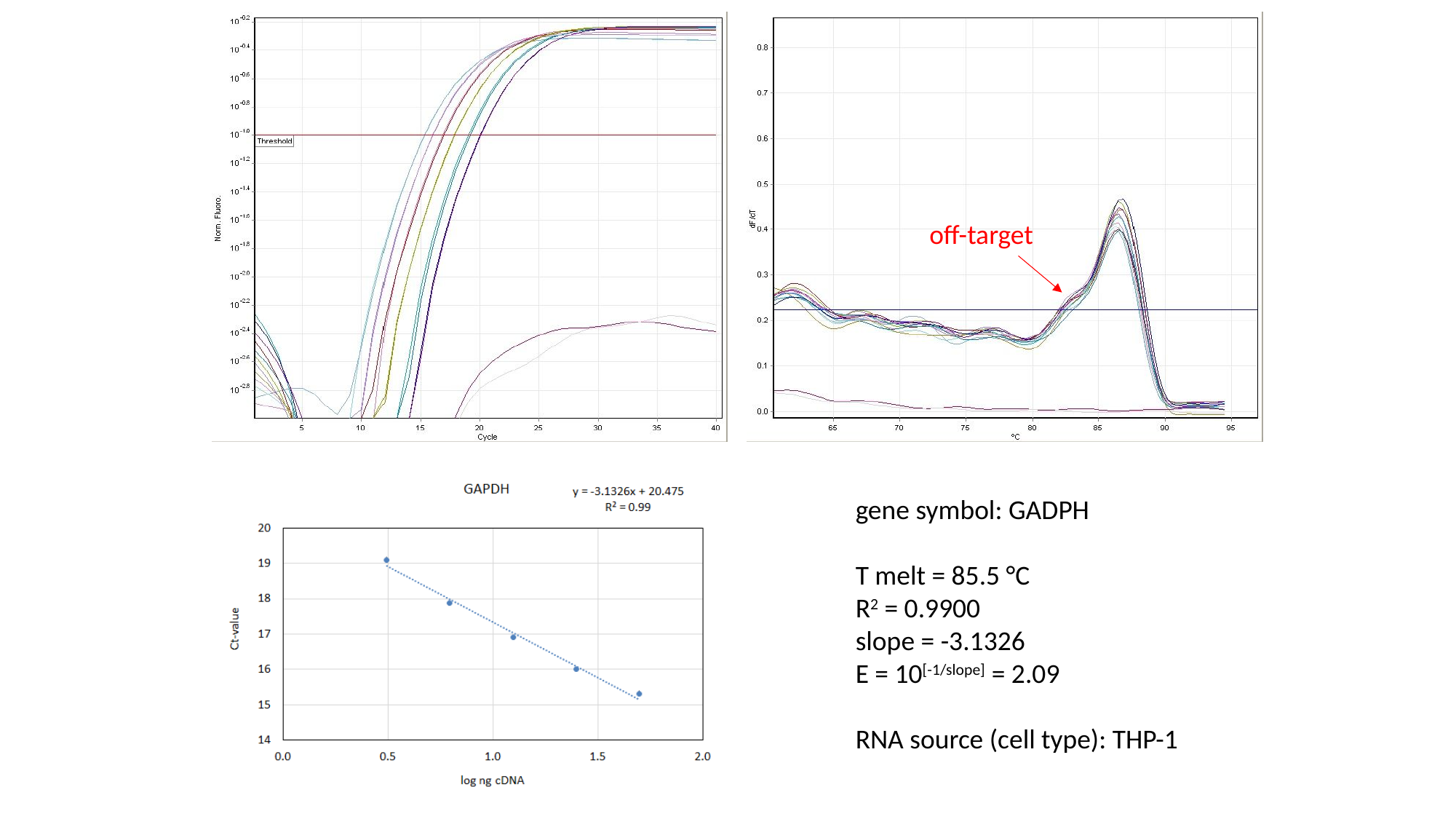

off-target
gene symbol: GADPH
T melt = 85.5 °C
R2 = 0.9900
slope = -3.1326
E = 10[-1/slope] = 2.09
RNA source (cell type): THP-1

## Slide 31
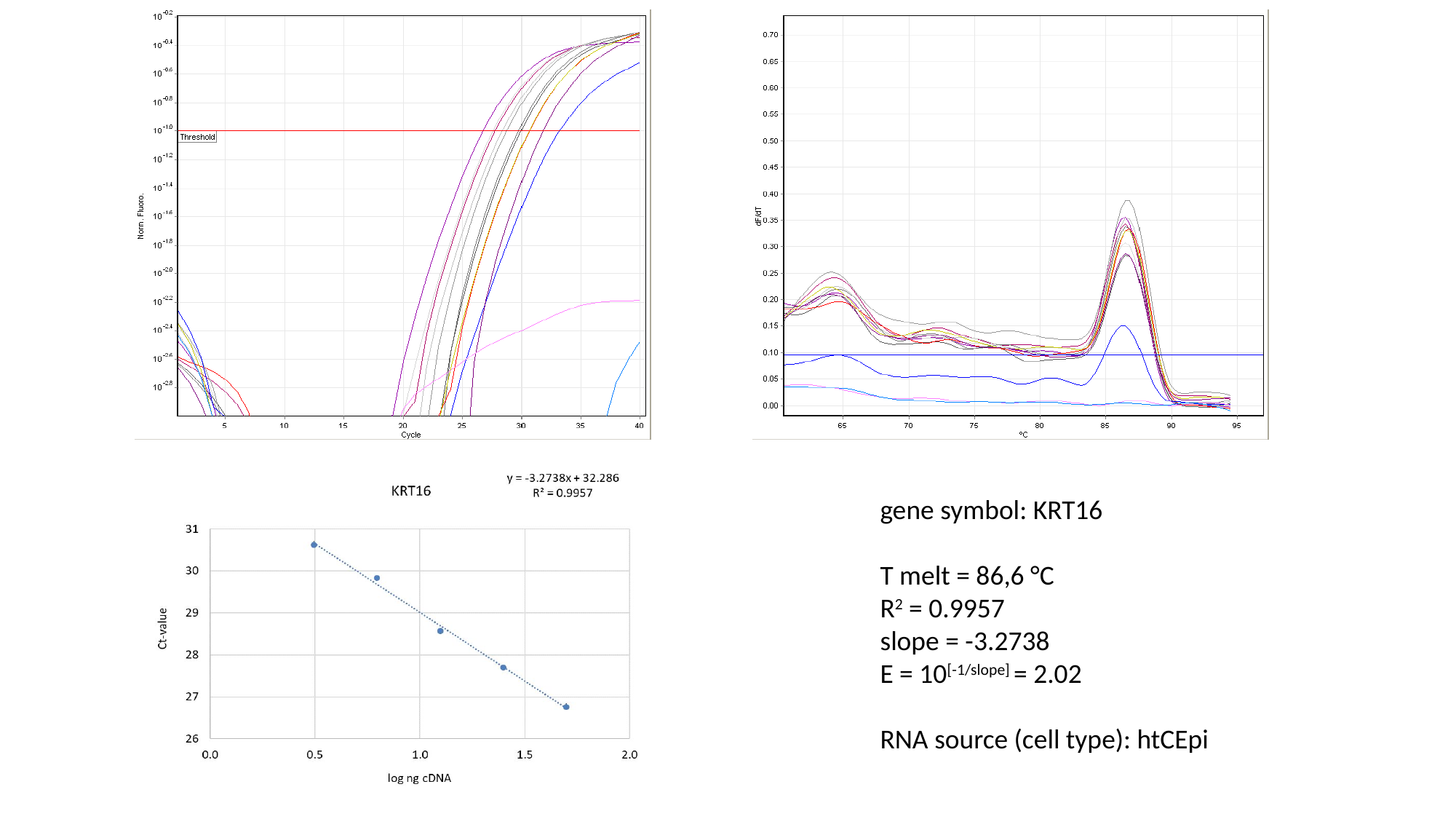

gene symbol: KRT16
T melt = 86,6 °C
R2 = 0.9957
slope = -3.2738
E = 10[-1/slope] = 2.02
RNA source (cell type): htCEpi

## Slide 32
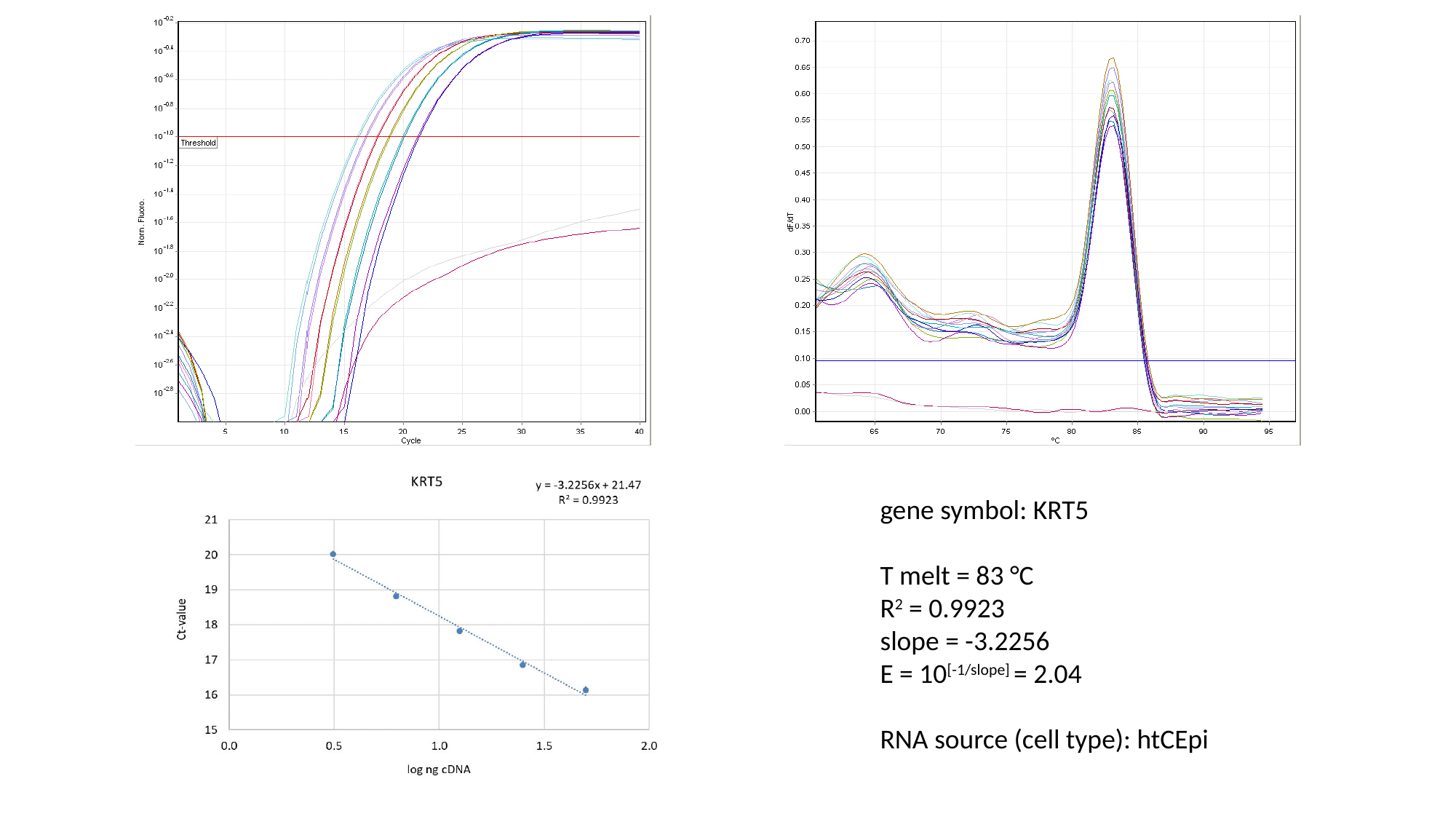

gene symbol: KRT5
T melt = 83 °C
R2 = 0.9923
slope = -3.2256
E = 10[-1/slope] = 2.04
RNA source (cell type): htCEpi

## Slide 33
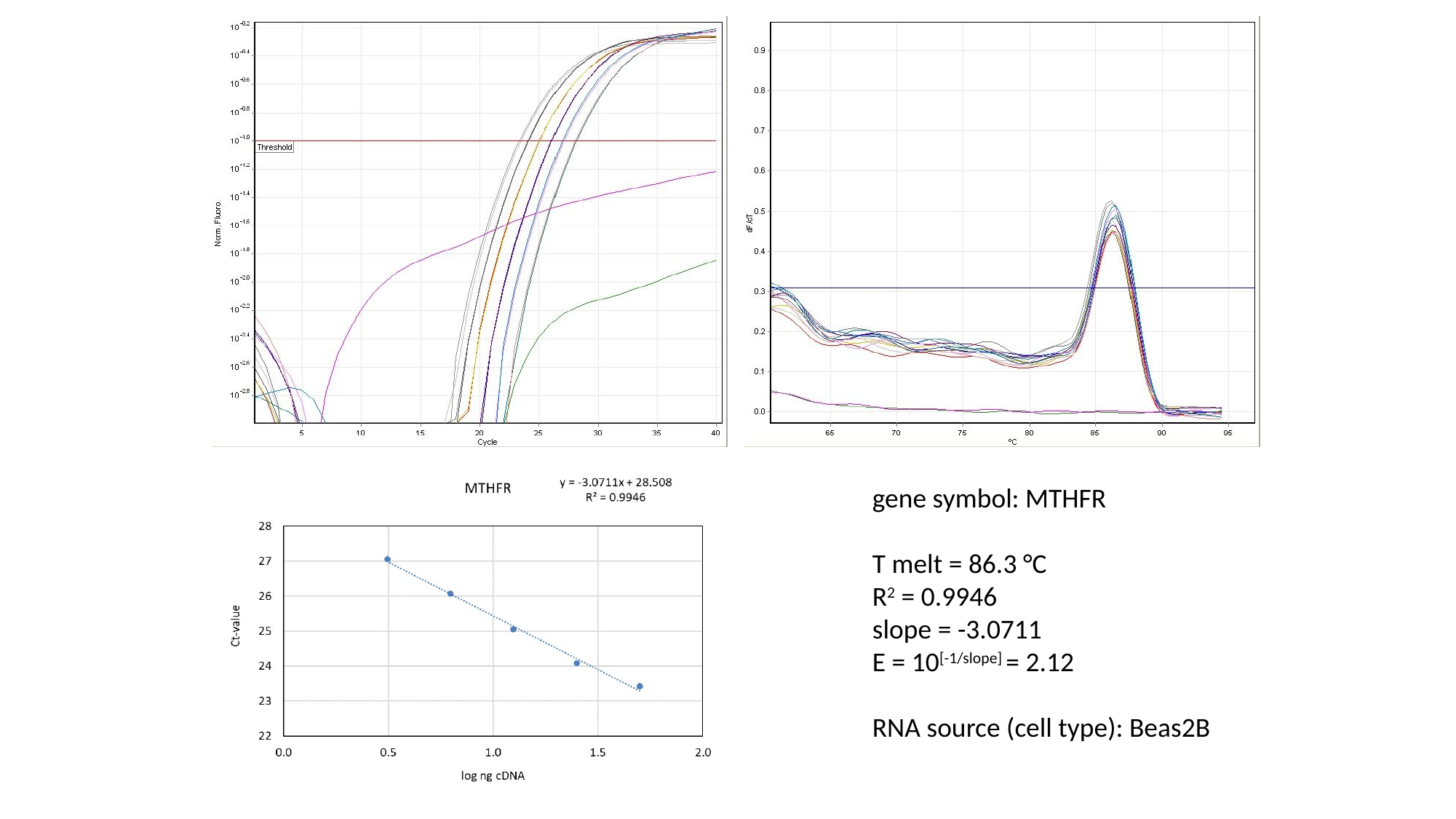

gene symbol: MTHFR
T melt = 86.3 °C
R2 = 0.9946
slope = -3.0711
E = 10[-1/slope] = 2.12
RNA source (cell type): Beas2B

## Slide 34
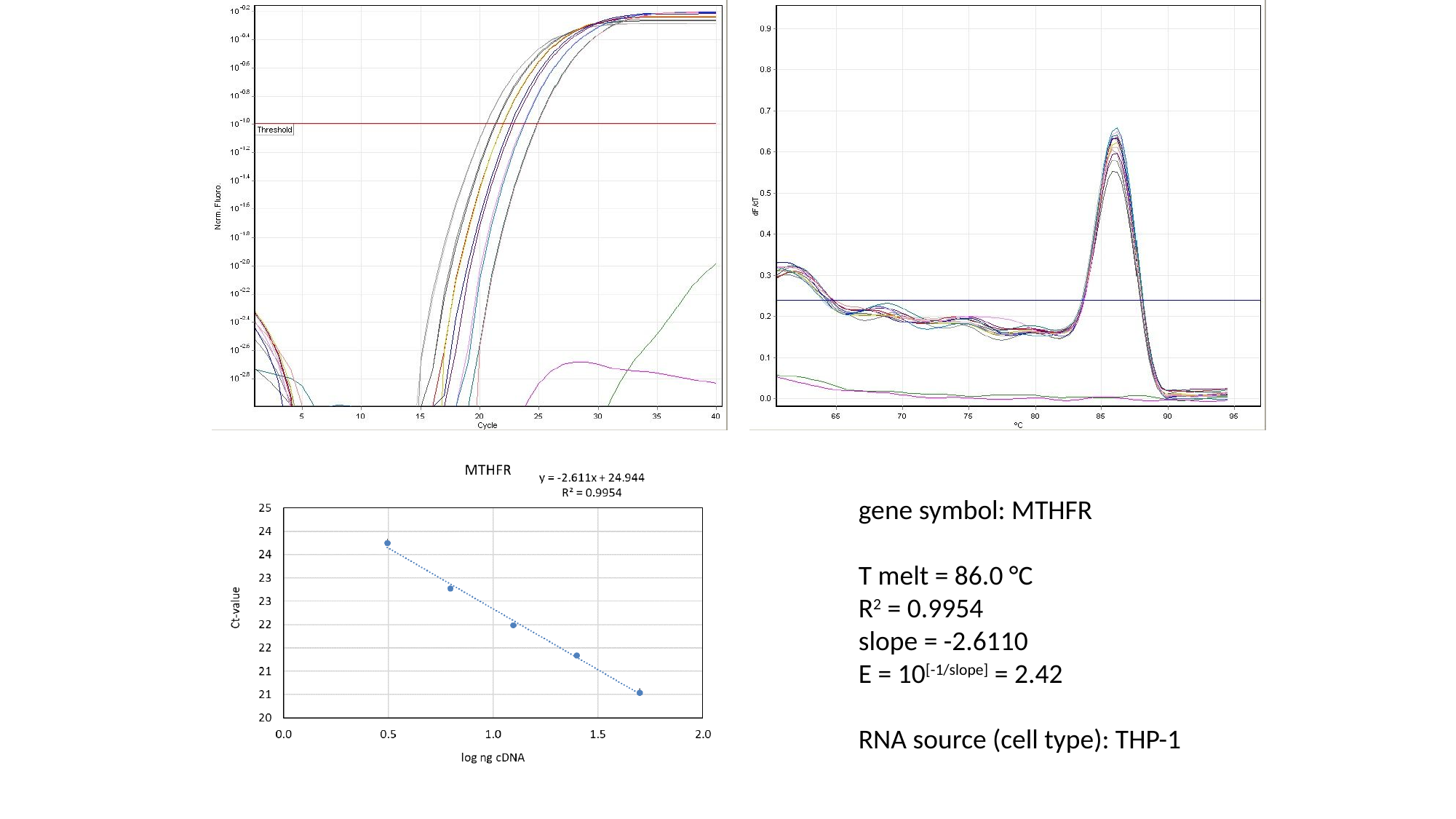

gene symbol: MTHFR
T melt = 86.0 °C
R2 = 0.9954
slope = -2.6110
E = 10[-1/slope] = 2.42
RNA source (cell type): THP-1

## Slide 35
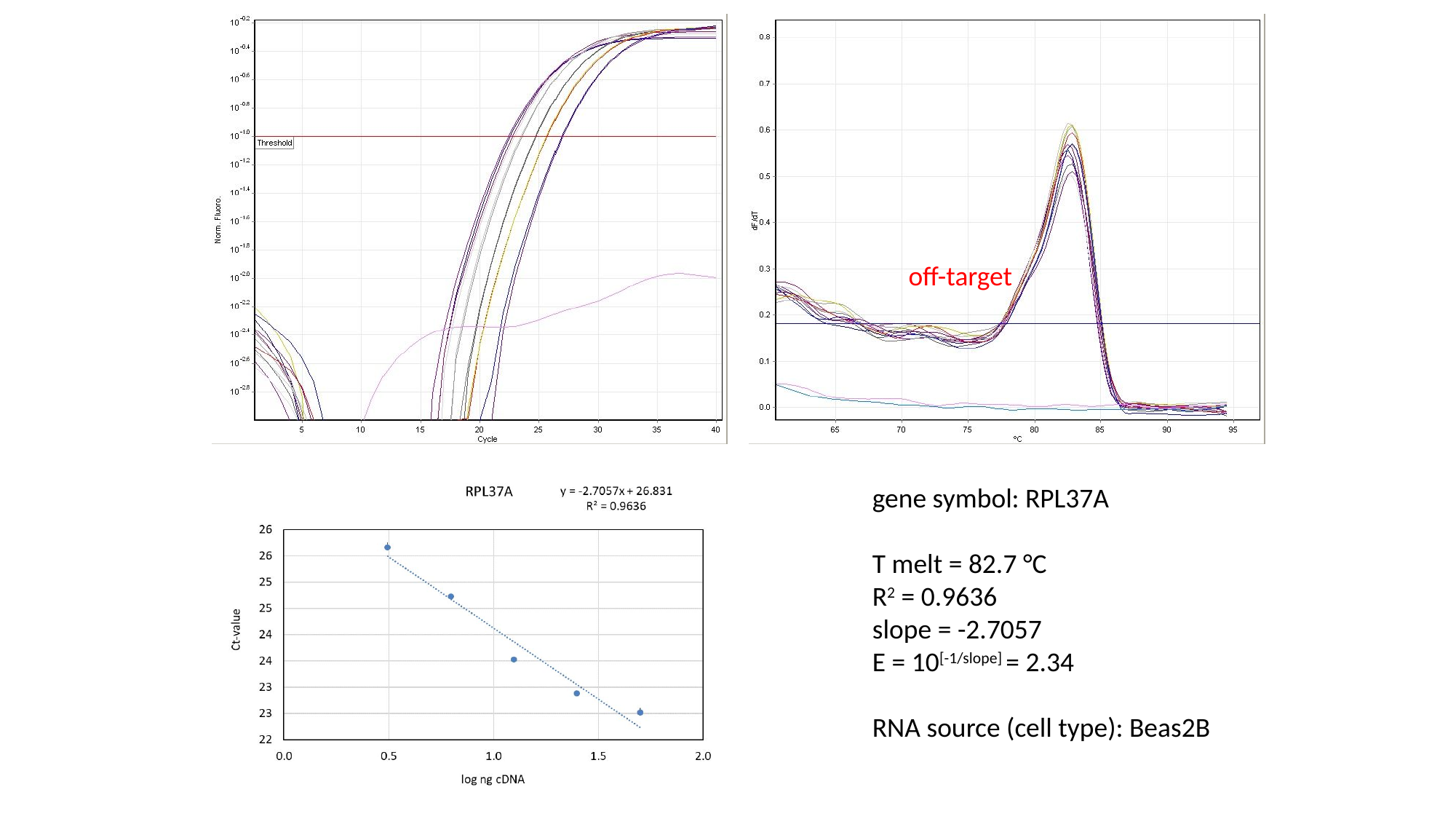

off-target
gene symbol: RPL37A
T melt = 82.7 °C
R2 = 0.9636
slope = -2.7057
E = 10[-1/slope] = 2.34
RNA source (cell type): Beas2B

## Slide 36
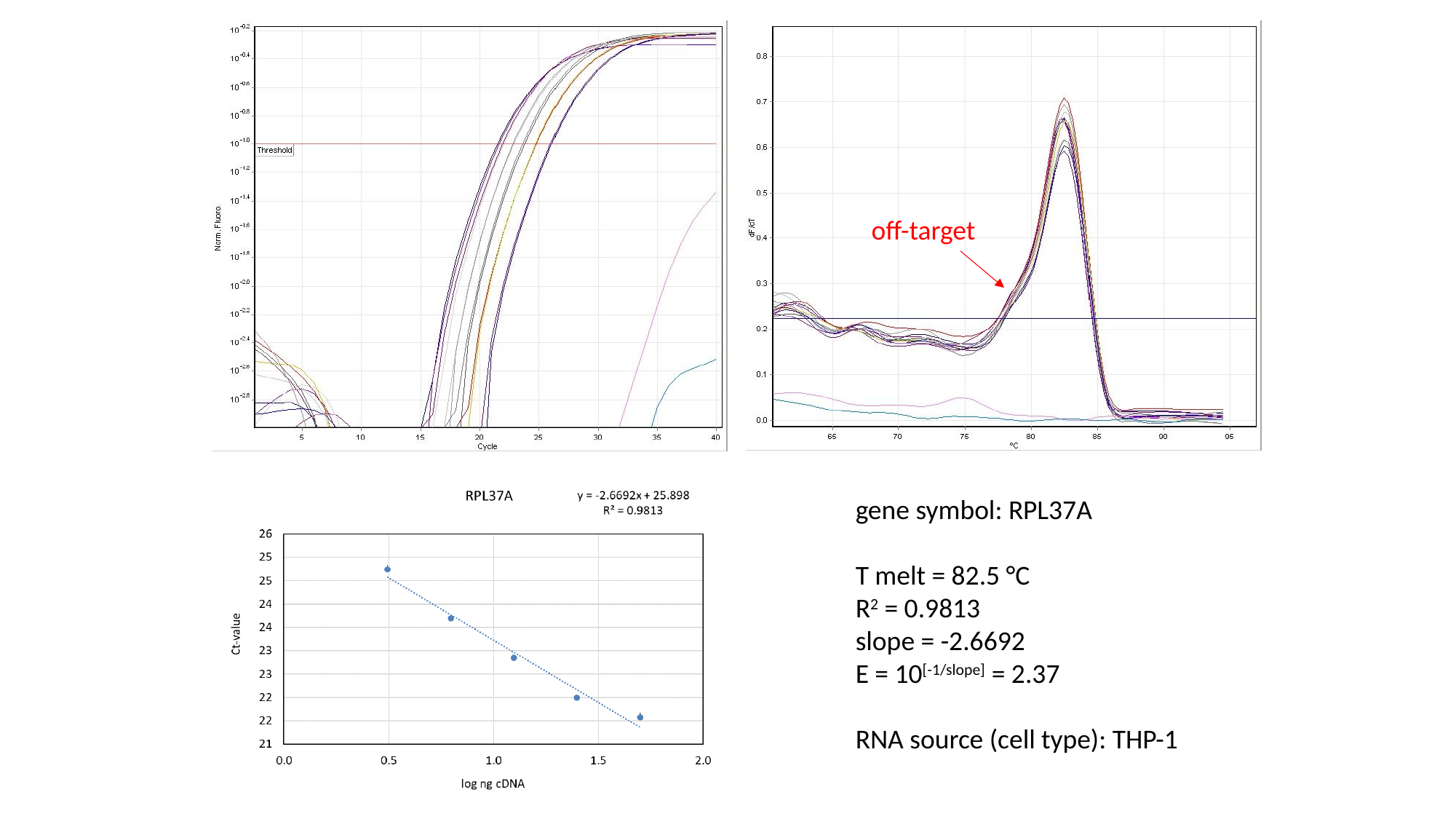

off-target
gene symbol: RPL37A
T melt = 82.5 °C
R2 = 0.9813
slope = -2.6692
E = 10[-1/slope] = 2.37
RNA source (cell type): THP-1

## Slide 37
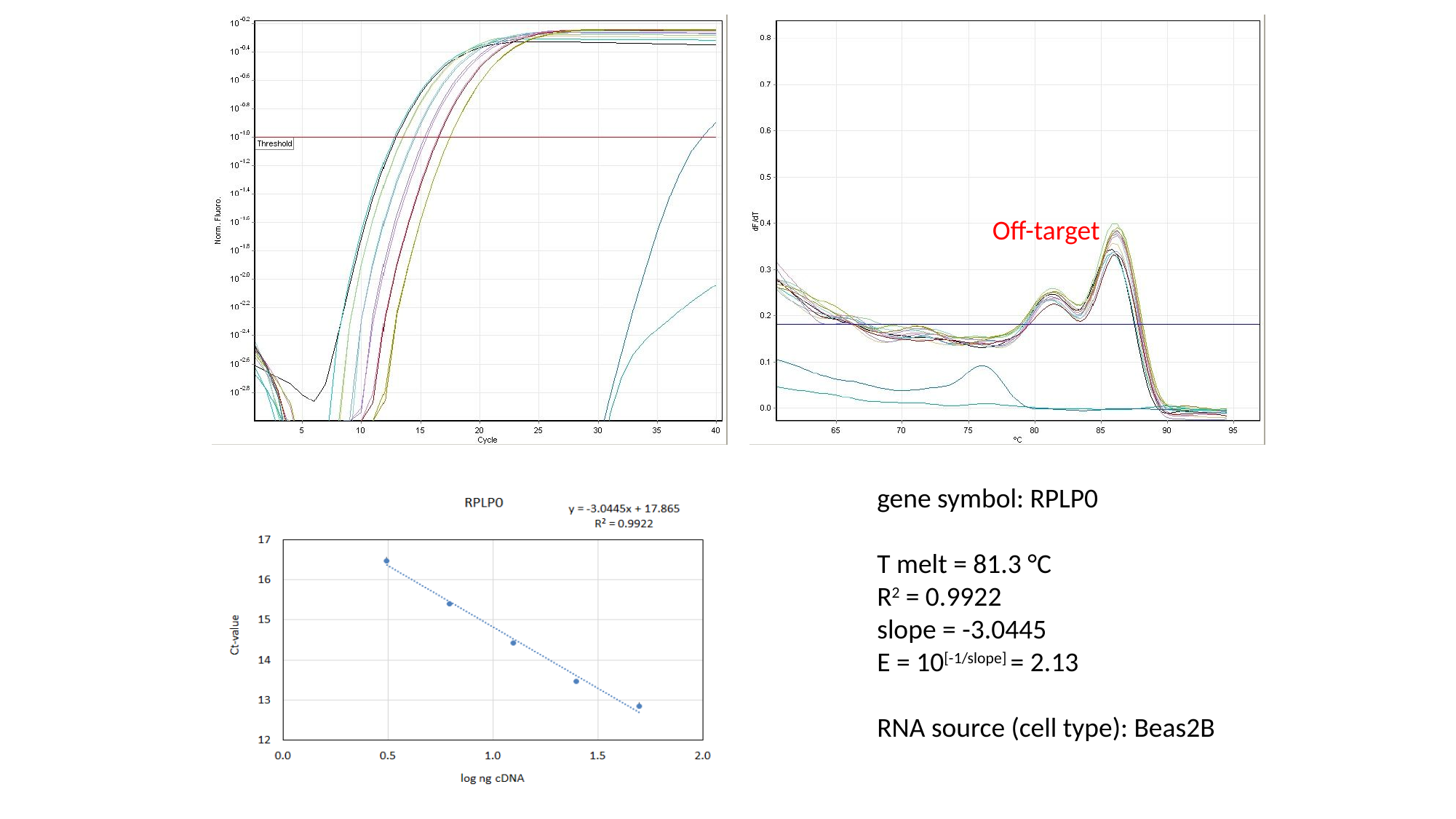

Off-target
gene symbol: RPLP0
T melt = 81.3 °C
R2 = 0.9922
slope = -3.0445
E = 10[-1/slope] = 2.13
RNA source (cell type): Beas2B

## Slide 38
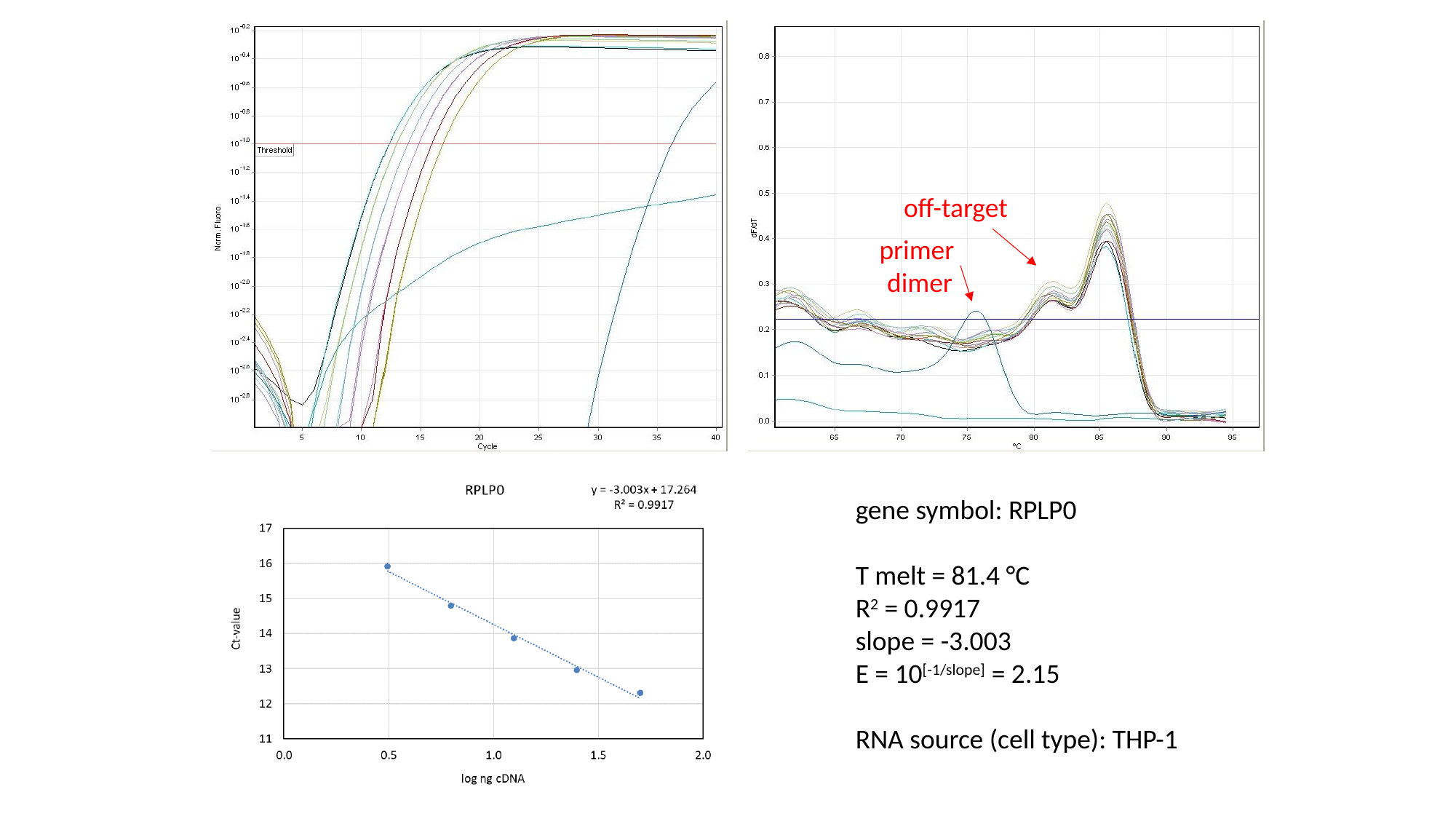

off-target
primer
dimer
gene symbol: RPLP0
T melt = 81.4 °C
R2 = 0.9917
slope = -3.003
E = 10[-1/slope] = 2.15
RNA source (cell type): THP-1

## Slide 39
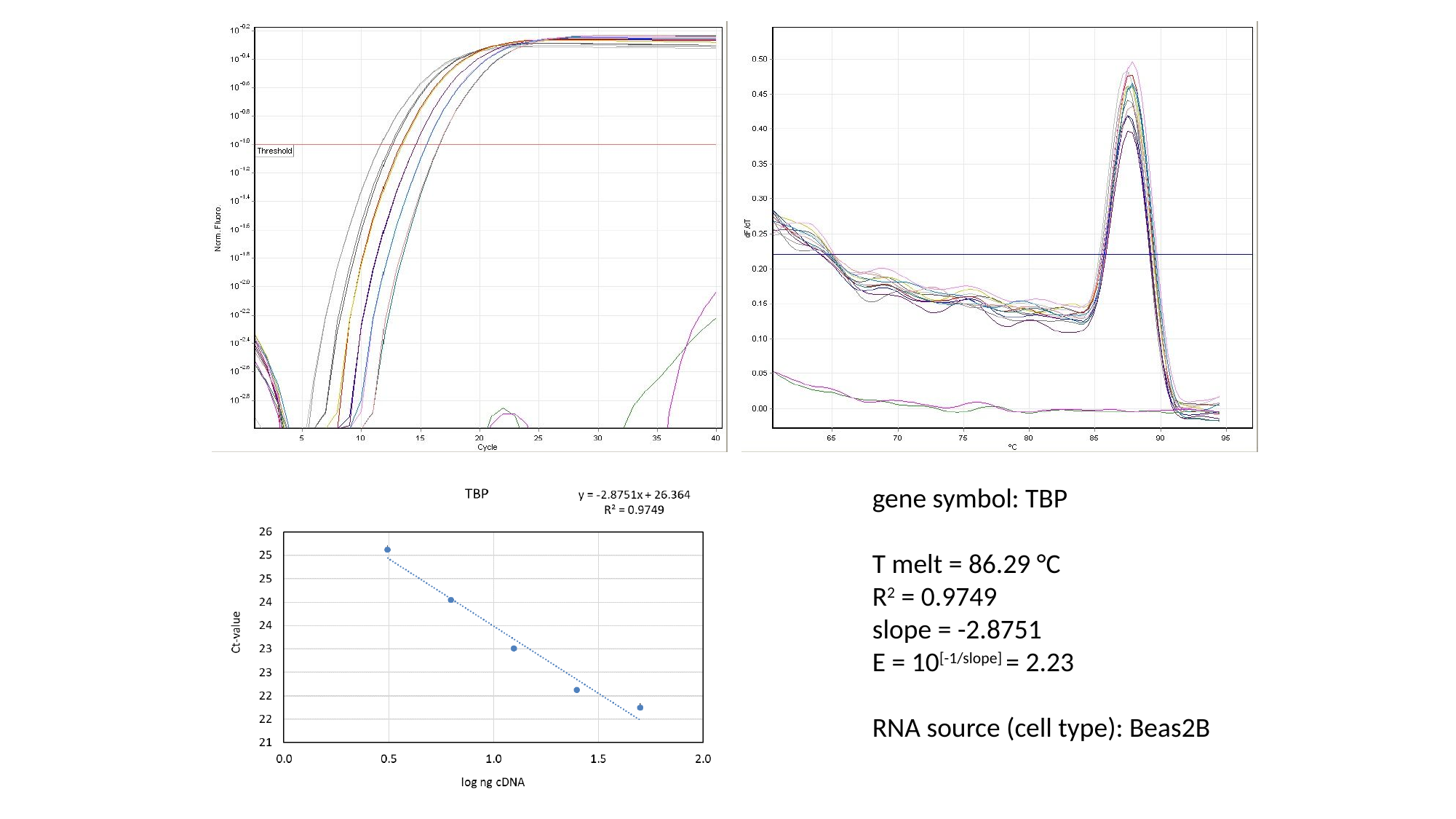

gene symbol: TBP
T melt = 86.29 °C
R2 = 0.9749
slope = -2.8751
E = 10[-1/slope] = 2.23
RNA source (cell type): Beas2B

## Slide 40
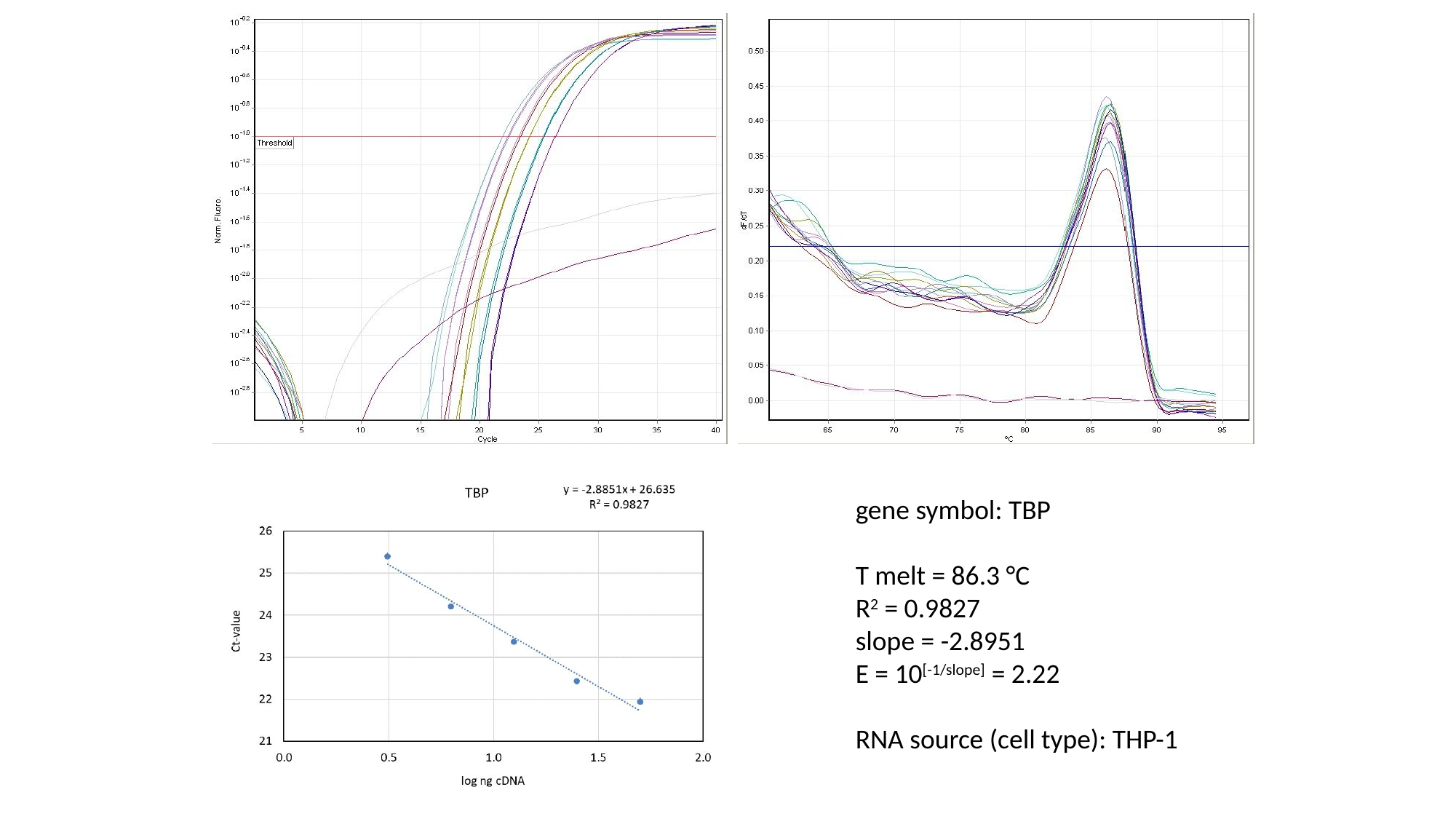

gene symbol: TBP
T melt = 86.3 °C
R2 = 0.9827
slope = -2.8951
E = 10[-1/slope] = 2.22
RNA source (cell type): THP-1

## Slide 41
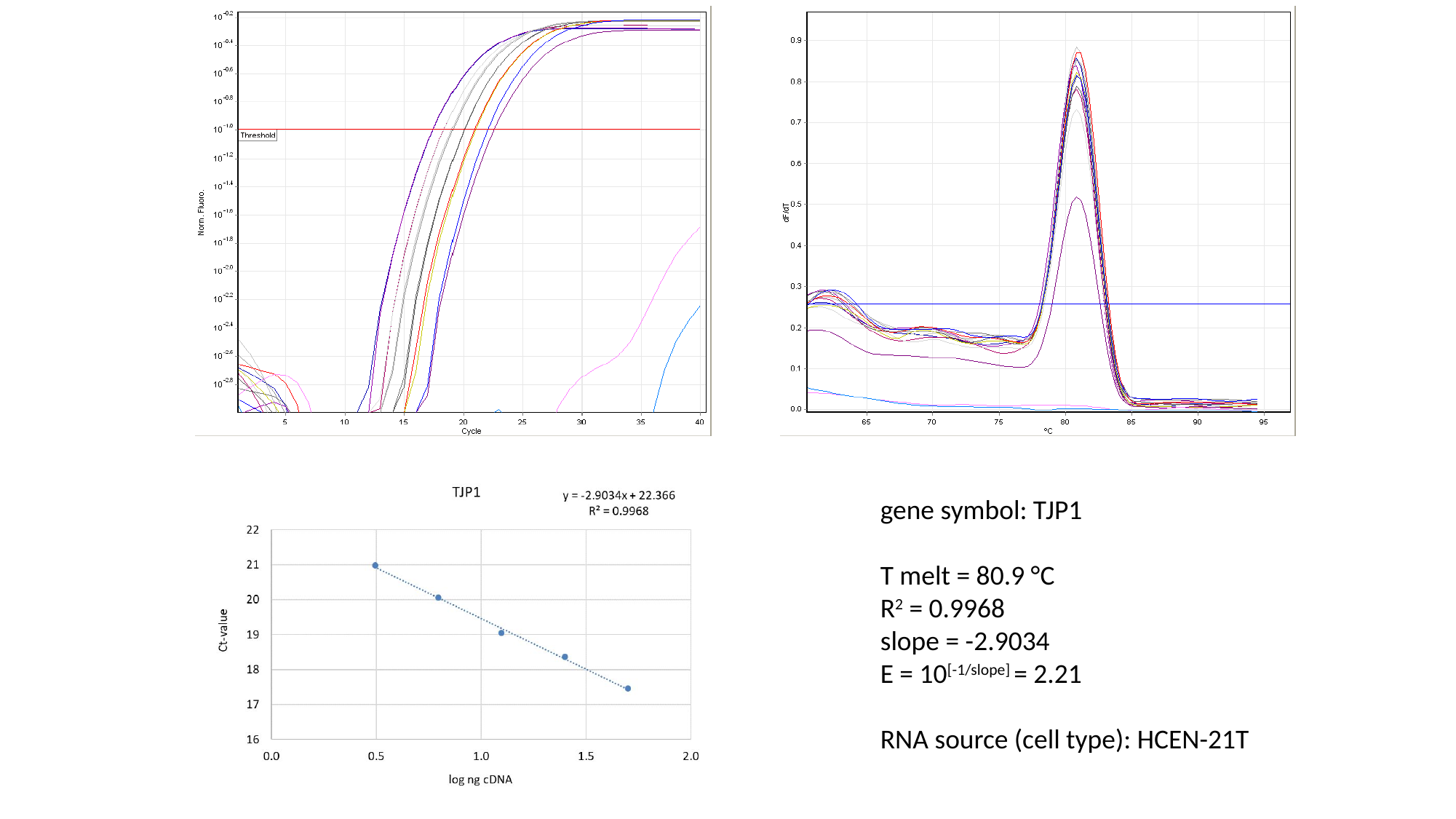

gene symbol: TJP1
T melt = 80.9 °C
R2 = 0.9968
slope = -2.9034
E = 10[-1/slope] = 2.21
RNA source (cell type): HCEN-21T
