# Supplementary figures and images for: ExonSurfer: a web-tool to design primers at exon–exon junctions
Source: BMC Genomics. 2024 Jun 12;25:594. doi: 10.1186/s12864-024-10456-2 (PMC11170769; doi:10.1186/s12864-024-10456-2)

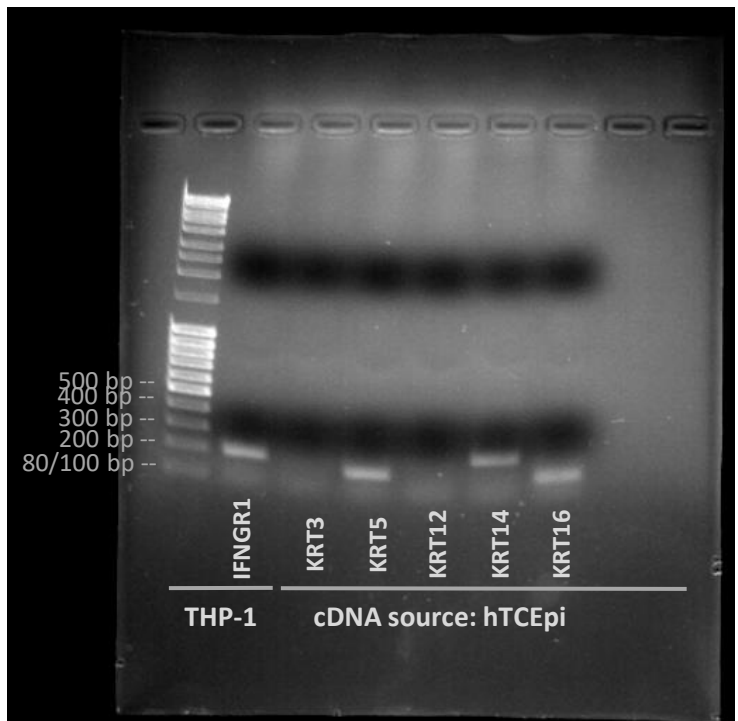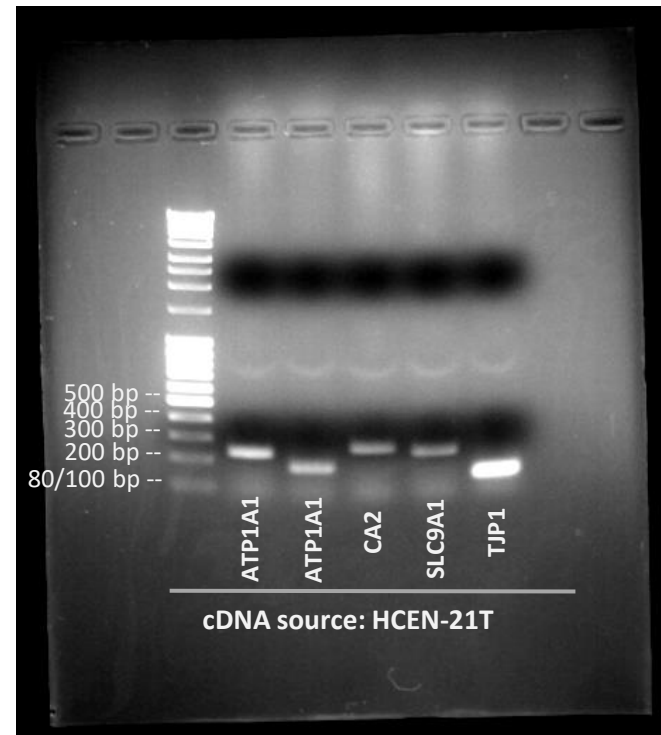

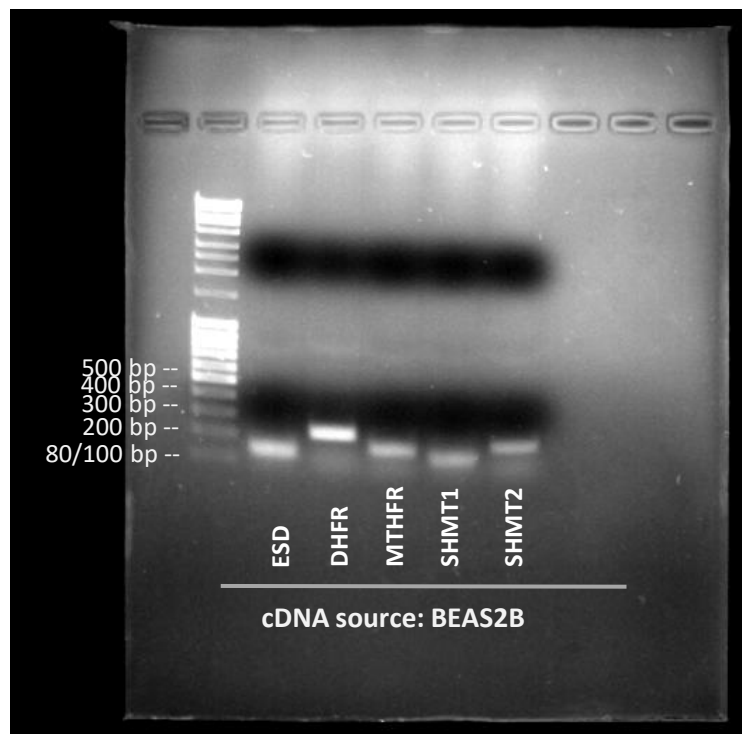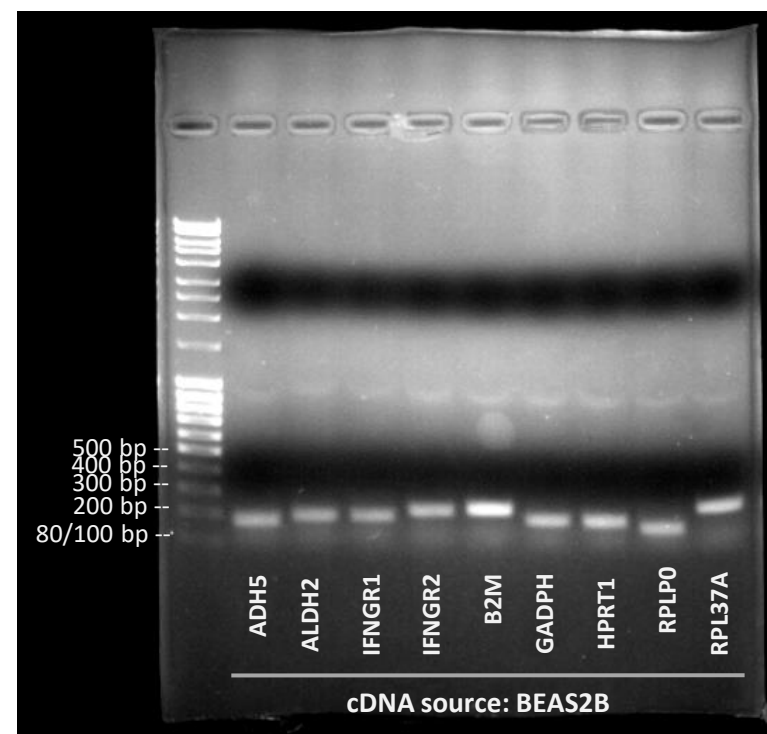

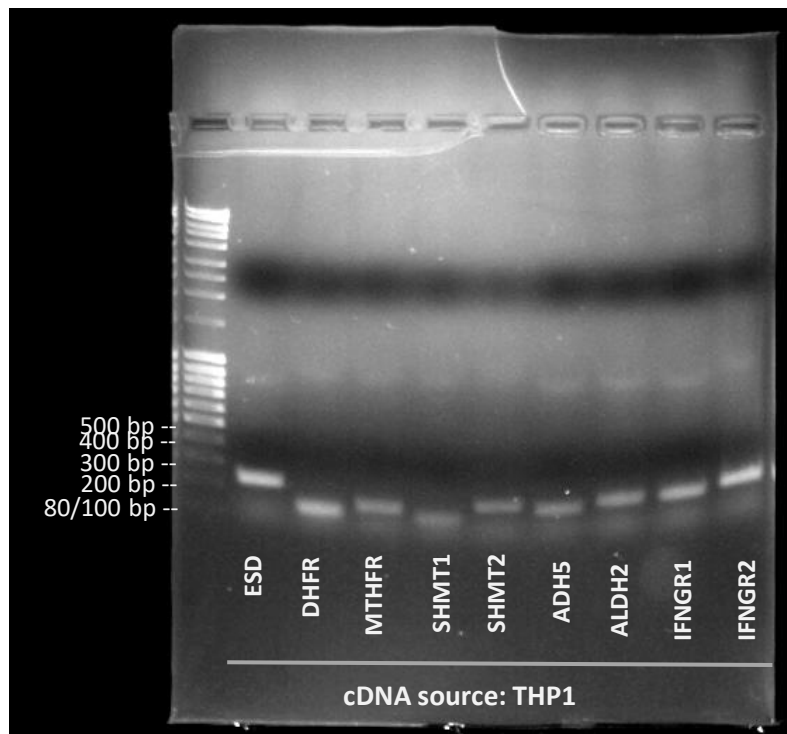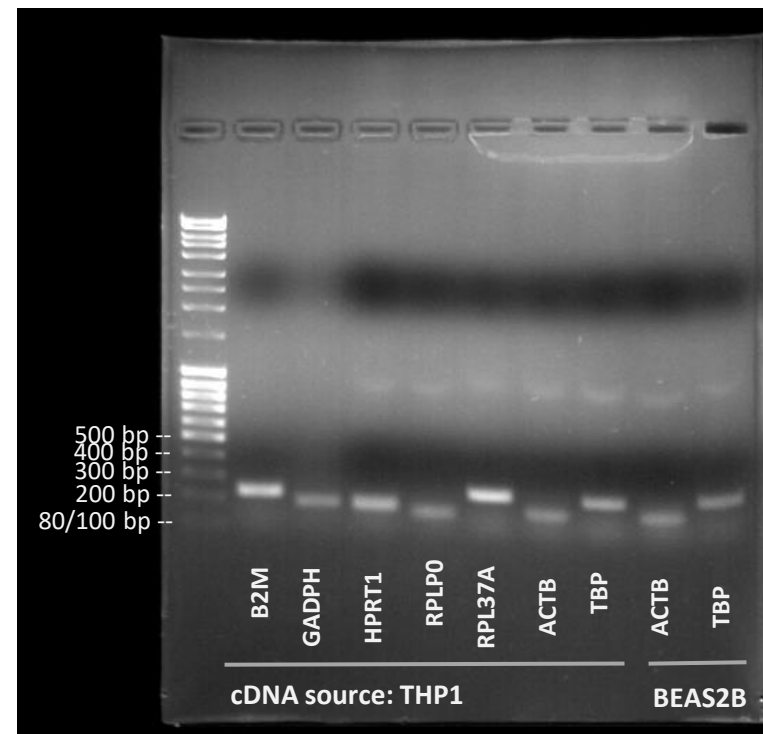

Supplement: Supplementary file 3 — Additional file 3: Supplementary File 3 displays electrophoresis gels containing the agarose gel-separated amplicons from the PCR products of the validation genes. [file 12864_2024_10456_MOESM3_ESM.pdf]
